# Type I IFNs facilitate innate immune control of the opportunistic bacteria *Burkholderia cenocepacia* in the macrophage cytosol

**DOI:** 10.1371/journal.ppat.1009395

**Published:** 2021-03-08

**Authors:** Michael G. Dorrington, Clinton J. Bradfield, Justin B. Lack, Bin Lin, Jonathan J. Liang, Tregei Starr, Orna Ernst, Julia L. Gross, Jing Sun, Alexandra H. Miller, Olivia Steele-Mortimer, Iain D. C. Fraser

**Affiliations:** 1 Signaling Systems Section, Laboratory of Immune System Biology, NIAID, NIH, Bethesda, Maryland, United States of America; 2 NIAID Collaborative Bioinformatics Resource, Frederick National Laboratory for Cancer Research, NIH, Frederick, Maryland, United States of America; 3 Salmonella-Host Cell Interactions Section, Laboratory of Bacteriology, NIAID, NIH, Hamilton, Montana, United States of America; Children’s Hospital Boston, UNITED STATES

## Abstract

The mammalian immune system is constantly challenged by signals from both pathogenic and non-pathogenic microbes. Many of these non-pathogenic microbes have pathogenic potential if the immune system is compromised. The importance of type I interferons (IFNs) in orchestrating innate immune responses to pathogenic microbes has become clear in recent years. However, the control of opportunistic pathogens–and especially intracellular bacteria–by type I IFNs remains less appreciated. In this study, we use the opportunistic, Gram-negative bacterial pathogen *Burkholderia cenocepacia* (*Bc*) to show that type I IFNs are capable of limiting bacterial replication in macrophages, preventing illness in immunocompetent mice. Sustained type I IFN signaling through cytosolic receptors allows for increased expression of autophagy and linear ubiquitination mediators, which slows bacterial replication. Transcriptomic analyses and *in vivo* studies also show that LPS stimulation does not replicate the conditions of intracellular Gram-negative bacterial infection as it pertains to type I IFN stimulation or signaling. This study highlights the importance of type I IFNs in protection against opportunistic pathogens through innate immunity, without the need for damaging inflammatory responses.

## Author summary

Our immune system protects us from infections caused by bacteria, viruses, fungi, and other organisms that we encounter regularly. The majority of these organisms have not evolved to cause us harm and are either tolerated within us or else are removed by the immune system. However, when the immune system is compromised, whether through illness, medication, or genetic abnormalities, some of these benign invaders become capable of causing significant, and even fatal, disease. These so-called ‘opportunistic pathogens’ are often difficult to treat due to antibiotic resistance and the fact that they infect people with compromised immune systems. In this study, we looked at how the sentinel cells of the immune system, called ‘macrophages’, can combat an opportunistic bacterial pathogen called *Burkholderia cenocepacia*, which infects patients with lung diseases like cystic fibrosis. We find that ancient immune proteins called interferons, which are often associated with battling viruses, are integral to macrophages’ ability to kill these bacteria, limiting the chance of severe disease. Macrophages produce interferons during and after engulfment of these bacteria, boosting their anti-bacterial killing machinery and priming neighboring cells to do the same. The results of the work could help in directing anti-*Burkholderia* treatments in susceptible patients.

## Introduction

Our immune system is not only important for the detection and elimination of pathogens, but also for surveying and manipulating the ever-changing non-pathogenic microbes that colonize our bodies, especially at mucosal sites. While a small proportion of microbial, viral, and fungal life is made up of ‘professional pathogens’, many more can cause disease in immunocompromised individuals [1–3]. A simple colonization event with these opportunistic pathogens can cause severe, and sometimes fatal, disease if the immune system is unable to perform its normal functions. Because these colonization events are so common, sentinel cells like macrophages are important for recognizing and neutralizing potential pathogens quickly while also limiting the potentially damaging effects of inflammation [4,5]. Understanding how innate immune responses to opportunistic pathogens protect healthy individuals can help us understand how to support immunity in immunocompromised states.

The *Burkholderia cepacia complex* is a group of bacteria containing, among others, the opportunistic pathogen *Burkholderia cenocepacia* (*Bc*), which is commonly associated with lung infections in patients with cystic fibrosis, chronic granulomatous disease, and other immunodeficiencies [6–8]. Upon inhalation, *Bc* is taken up by alveolar macrophages but avoids killing by escaping the phagosome and replicating in the cytosol [9–12]. *Bc* is also capable of subverting the autophagy response, leading to a rapid replication in these cells [12]. Perhaps most importantly, *Bc* is a soil-dwelling bacterium that is highly resistant to antibiotics, making it very difficult to treat in immunocompromised individuals. Because *Bc* does not cause illness in immunocompetent people despite common periodic colonization, it is a good candidate for studying innate immune responses to opportunistic pathogens that have not evolved solely to infect humans.

The type I interferons (IFNs) are a family of cytokines commonly associated with protecting the host against viral infections. A subset of these proteins is produced by the majority of cell types in the body and all act through binding to the heterodimeric IFNα/β receptor (IFNAR), which is made up of two chains: IFNAR1 and IFNAR2 [13]. When IFNAR recognizes its ligand, a signaling cascade culminates in the activation of members of the signal transducer and activator of transcription (STAT) family of transcription factors. This leads to the transcription of a cluster of genes termed ’IFN-stimulated genes’, or ISGs. These ISGs are responsible for the anti-viral and anti-proliferative states associated with IFN production during viral infections and interferonopathies [13–17]. Type I IFNs are produced by many cell types including epithelial cells, macrophages, and dendritic cells. Unlike the type II IFN, IFNγ, type I IFNs are part of the initial phase of innate immunity and do not require adaptive immune activation to reach therapeutic levels in the body during infection.

Bacterial infection can lead to the production of IFNs downstream of multiple pattern recognition receptors (PRRs) found on macrophages, including Toll-like receptors (TLRs) [18–20], RIG-I-like receptors (RLRs) [16], and other cytosolic nucleic acid sensors such as MAVS and STING [21]. While the importance of IFN signaling in the context of viral infection is well-established, there is less information regarding the role of these cytokines during bacterial infection. However, the various links between evolutionarily conserved bacteria-sensing PRRs and IFN production implies an important role for IFNs during these infections. Contextual clues point to IFN being especially important during intracellular infections [22,23]. For example, many of the PRRs listed above reside in the cytoplasm of cells, therefore requiring their ligands have access to this intracellular space. Additionally, triggering of IFN production downstream of TLR4 first requires the endocytosis of TLR4 and the subsequent recruitment of the signaling adaptor TRIF [19,24]. This direct link between the sensing of a common bacterial ligand (in this case lipopolysaccharide, or LPS) within the endosome and the production of IFNs heavily implicates IFN in the protection of macrophages from bacterial invasion. Whether *Bc* infection induces a robust IFN response in macrophages, or how IFN signaling affects cytosolic *Bc* replication have not been previously investigated.

In this study, we looked at how type I IFNs support cell-intrinsic anti-*Bc* immunity in macrophages. *In vivo* studies show that, unlike during LPS shock experiments wherein IFN signaling is detrimental to the host, alveolar macrophages in *Ifnar1*^-/-^ mice are more susceptible to bacterial replication, leading to significant illness compared with WT mice. Prophylactic IFNβ, but not IFNγ, is capable of inducing an anti-bacterial state in WT macrophages, limiting the amount of bacterial replication in these cells, likely due to increases in ubiquitin deposition and autophagy of the bacteria. Sustained type I IFN production and downstream signaling are vital to protect macrophages from *Bc* infection, and do not rely on the endosomal TLR4-TRIF signaling axis of bacterial IFN induction, instead depending on cytosolic signaling through MAVS and STING. We also show significant differences in the transcriptional response to *Bc* compared to LPS stimulation alone. These studies highlight the role of multiple macrophage PRRs in stimulating type I IFN responses to bacterial infection and how these cytokines allow for innate immune protection against the opportunistic pathogen *Bc*.

## Results

### Type I IFN signaling protects mice from morbidity due to *Burkholderia* infection

**Fig 1:**
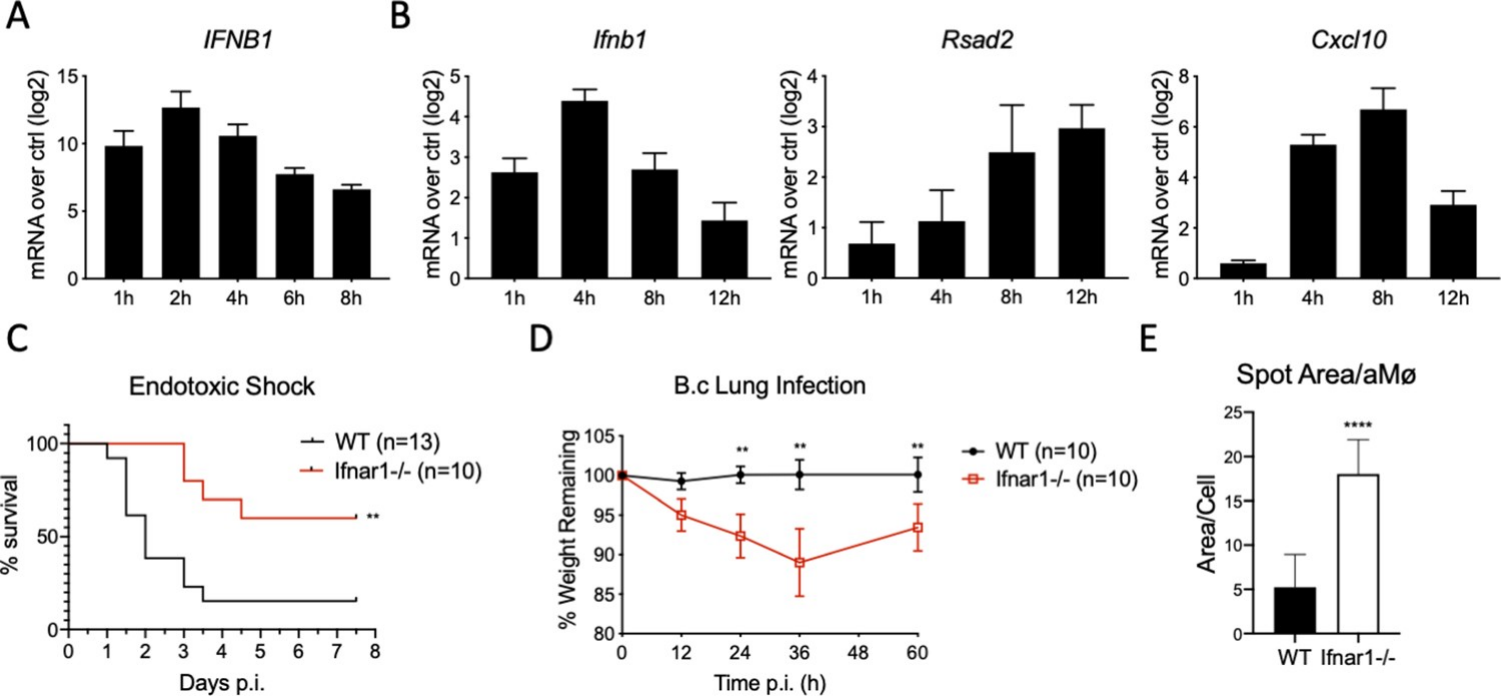
*IFN signaling has disparate effects on* in vivo *LPS stimulation and Gram-negative bacterial infection*. A) Differentiated THP-1 cells and B) BMDMs were infected with live *Bc* for the indicated times before RNA extraction and RT-PCR for given genes. Data show relative mRNA expression to unstimulated cells using *Hprt* as housekeeping gene; n = 5, representative of two experiments C) Littermate control WT (n = 13) and *Ifnar1*^-/-^ (n = 10) mice were injected intraperitoneally with 10 mg/kg of LPS and mice were weighed twice a day for 7 days. D, E) Lightly anesthetized, littermate control WT (n = 10) and *Ifnar1*^-/-^ (n = 10) mice were infected with 5x10^6^ CFUs of *Bc* via intranasal instillation. D) Mice were weighed every 12 h for 5 days. E) Broncho-alveolar lavages were performed 16 h post-infection and bacteria were enumerated using high-content imaging; all *in vivo* experiments are shown as a combination of two independent experiments C) ** = p≤0.01 by Mantel-Cox test; D, E) ** = p≤0.01, **** = p≤0.0001 by Mann-Whitney U test.

In order to ensure that the LPS-TLR4-IFN paradigm found for many Gram-negative pathogens holds during cellular infection with *Burkholderia cenocepacia*, we infected differentiated THP-1 cells, a human monocyte/macrophage cell line (Fig 1A), as well as murine bone marrow-derived macrophages (BMDMs, Fig 1B) with J2315, a common laboratory strain of *Bc*, and measured *IFNB1/Ifnb1* transcription (Fig 1A and 1B) as well as that of the canonical ISGs *Rsad2* and *Cxcl10* (Fig 1B). Infection of both human and murine macrophages resulted in the robust upregulation of the IFNβ gene, followed by the subsequent upregulation of ISGs, showing a significant type I IFN response in these cells when infected with *Bc*.

The process of type I IFN production by macrophages downstream of bacterial stimulation is well-established, especially for infections with Gram-negative bacteria wherein recognition of LPS by endosomal TLR4 leads to the activation of interferon regulatory factor 3 (IRF3) [19,24]. However, the *in vivo* contributions of type I IFNs to host responses to bacterial infection are much more complicated. Even before the mechanistic link between LPS stimulation and IFN production was solidified, multiple groups attempted to determine a role for type I and type II IFNs in either reducing or exacerbating the effects of acute endotoxic shock in mice [25–29]. The results of these studies, taken together, have been inconclusive, however most show an increase in mortality in the presence of intact IFN signaling [26–28], with the ISG *Ifit2* being especially important in this regard [29]. What has not been reported to date, is a properly controlled endotoxic shock trial involving age-, sex-, and (most importantly) litter-matched WT and *Ifnar1*^-/-^ mice exposed to a lethal dose of LPS. Upon performing this experiment, we found that there was a significant and repeatable decrease in mortality in the *Ifnar1*^-/-^ mice compared to their WT littermates, indicating a negative impact to the host for IFN signaling in this widely-used experimental model (Fig 1C). One should note that if mice that were not littermate-controlled were used, the outcome of this experiment varied considerably, supporting the importance of using properly controlled groups of mice for these types of experiments (S1A and S1B Fig). This also points to the potential importance of the microbiome and/or other environmental variables in regulating type I IFN-dependent inflammatory responses.

Having established a negative role for IFNs in the context of endotoxic shock, the question remained as to whether the insights gleaned from these PAMP-centric experiments could be extrapolated to a more complex stimulus, such as a Gram-negative bacterial infection. To this end, we intra-nasally infected WT and *Ifnar1*^-/-^ mice with 5x10^6^ CFUs of dsRed+ J2315. While WT mice showed no clinical signs of infection, including no weight loss whatsoever compared to pre-infection, *Ifnar1*^-/-^ mice showed appreciable weight loss through the first few days of infection, with weight loss peaking at 10–15%, followed by a gradual return to health (Fig 1D). This was despite similar levels of cytokines and chemokines in the broncho-alveolar lavages of these mice (S1C Fig). The lack of clinical signs in WT mice infected with such a high dose of bacteria directly in the lungs was striking, and points to the robustness of the innate immune response to these bacteria in healthy animals. To test if the phenotype found in *Ifnar1*^-/-^ mice could be a result of bacterial proliferation within alveolar macrophages, we removed alveolar macrophages from mice infected for one day and imaged them to enumerate the bacterial load in these cells. We found that *Ifnar1*^-/-^ macrophages harbored significantly greater levels of bacteria, as measured by dsRed fluorescence, potentially indicating a decrease in the microbicidal activity of these cells compared to their WT counterparts (Fig 1E). This led us to interrogate the impact of type I IFN signaling on macrophage responses to *Bc in vitro*.

### Type I, but not type II, IFN signaling in macrophages protects them from intracellular replication

**Fig 2:**
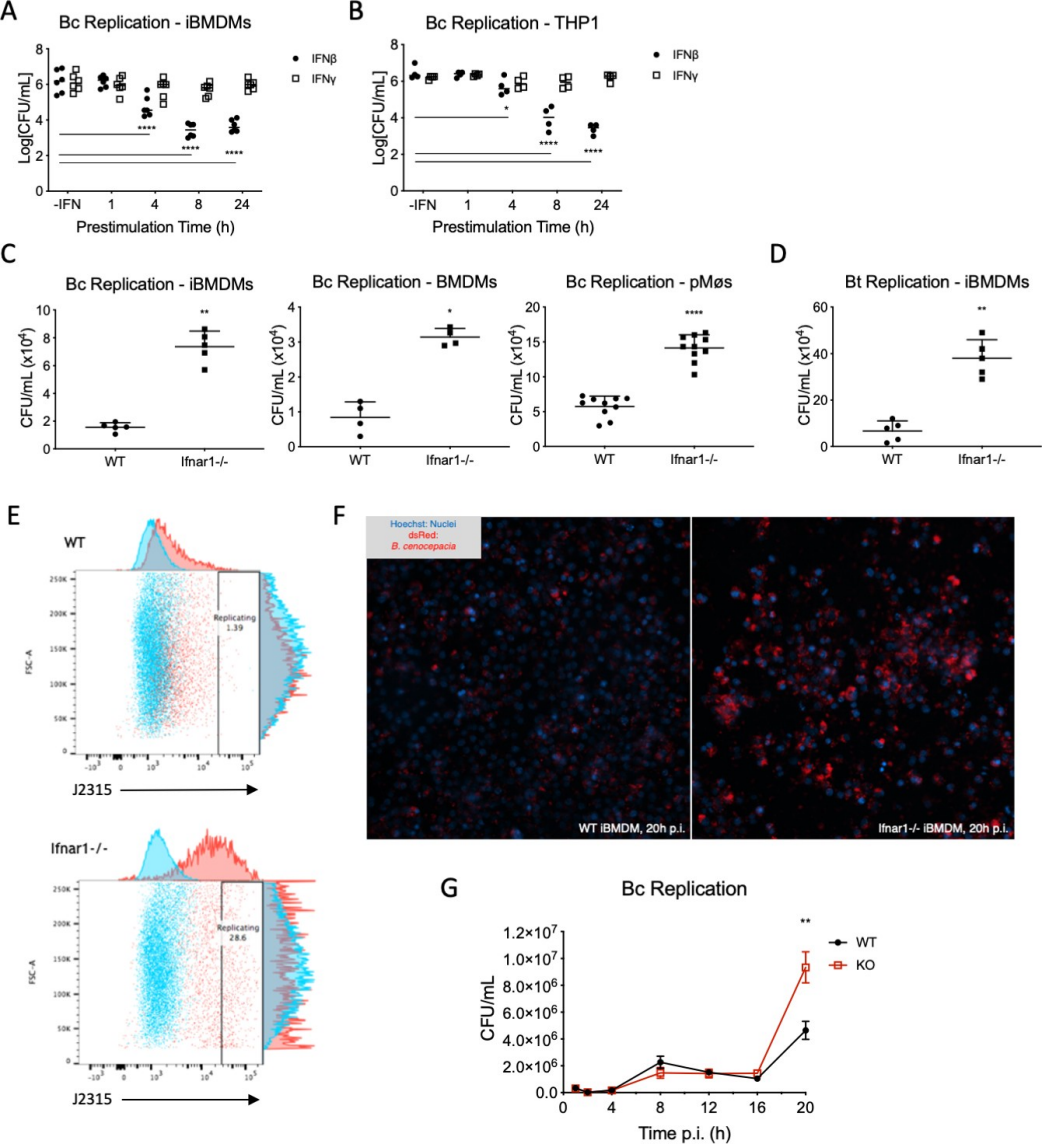
Burkholderia *infection stimulates a robust type I IFN response in macrophages*, *protecting them from intracellular replication*. A) iBMDMs or B) differentiated THP-1 cells were pre-treated with recombinant IFNβ or IFNγ for the indicated times, after which media was replaced and cells were infected with *Bc* (MOI = 1) for 22 h. Cells were lysed and lysates were cultured for 24 h before enumerating colonies; n = 5 per condition, representative of 3 independent experiments. C, D) WT and *Ifnar1*^-/-^ macrophages were infected with C) *Bc* (MOI = 1) or D) *Burkholderia thailandensis* (MOI = 1) for 22h before cells were lysed. Lysates were then cultured and bacterial colonies were enumerated after 24 h of growth. E, F) WT and *Ifnar1*^-/-^ iBMDMs were infected with live dsRed+ *Bc* (MOI = 1) and fixed in 2% PFA after 22 h. Bacterial load was analyzed using E) flow cytometry or F) high-content imaging based on dsRed fluorescence. G) WT and *Ifnar1*^-/-^ iBMDMs were infected with wt *Bc* (MOI = 1) for the indicated times before being lysed. Lysates were plated, as in 2C, and bacterial colonies were counted after 24 h of culture on blood agar plates. All *Bc* replication experiments above had n ≥ 5 and are representative of ≥ 3 experiments. * = p≤0.05, ** = p≤0.01, and **** = p≤0.0001 by Mann-Whitney U test.

The link between TRIF signaling and IFNβ production has been conserved through evolution, with TRIF analogues dating back to the emergence of jawed fish [30]. This conservation suggests a great importance for IFN production downstream of bacterial infection, and we hypothesized that autocrine and paracrine signaling through the type I IFN receptor would lead to a protective phenotype in macrophages infected with *Bc*. However, type I IFNs are not the only IFNs with antibacterial activity. Certainly, one of the primary roles of adaptive immunity, in the context of bacterial infection, is the production of IFNγ, the sole type II IFN, which stimulates the upregulation of various antimicrobial pathways in macrophages [31]. The ability of macrophages to kill many bacterial species is augmented by IFNγ treatment, and both mice and humans who lack proper IFNγ activity are susceptible to recurrent and sometimes fatal bacterial infections [31–34]. To test whether IFNβ and/or IFNγ were able to protect macrophages from *Bc* replication, we performed a timecourse of IFN pre-stimulation with each of the cytokines before infecting macrophages with J2315 for 22 hours before lysing the cells and plating serial dilutions of the lysate on blood agar in order to enumerate the colony-forming units (CFUs). Pre-stimulation of both immortalized mouse bone marrow-derived macrophages (iBMDMs) (Fig 2A) and differentiated THP-1 cells (Fig 2B) with IFNβ for at least 8h led to a reduction in bacterial CFUs from lysed cells. Surprisingly, pre-stimulation with IFNγ showed no such result, implying that IFNγ stimulation did not increase the anti-microbial activity in these cells. This again points to the ability of innate immunity to deal with these potential pathogens without the aid of cytokines associated with adaptive immune responses.

Having established a prophylactic effect for exogenous IFNβ treatment of macrophages infected with *Bc*, we sought to test how bacterial replication was affected by a lack of type I IFN signaling in these cells. Firstly, we infected various macrophage populations, including BMDMs, peritoneal macrophages (pMøs), and iBMDMs, with J2315 before lysing the cells and plating the lysates, as above. These experiments show that, in all three cell types, macrophages lacking IFNAR1 harbored more bacteria than their WT counterparts (Fig 2C). This was also true of iBMDMs infected with the closely related bacterial species *B*. *thailandensis*, showing the cells’ dependence on IFN signaling was not specific to *Bc* infection (Fig 2D). Secondly, we infected iBMDMs with either a strain of J2315 expressing a plasmid containing the fluorophore dsRed or a GFP-expressing *B*. *thailandensis* and measured bacterial load by flow cytometry (Figs 2E and S2A). Again, *Ifnar1*^-/-^ iBMDMs allowed for more robust bacterial replication than WT cells, as shown by increased bacterial fluorescence in these cells at 22 hours post-infection. Finally, we used high-content imaging to visualize these dsRed+ bacteria in WT and *Ifnar1*^-/-^ BMDMs (Fig 2F). Despite counting the same number of total cells (S2B Fig), spot counting analysis showed a significant increase in *Bc*-containing spots per *Ifnar1*^-/-^ macrophage compared to WT cells (Figs 2F and S2C). There were, however, fewer cells per field despite equal seeding numbers (S2D Fig), suggesting that *Ifnar1*^-/-^ cells could be dying at a more rapid rate than their WT counterparts. This was confirmed by a glucose 6-phosphate dehydrogenase release assay (S3A Fig). Concurrent to these high-content imaging experiments, we ran a timecourse of infection wherein cells were collected at various timepoints, lysed, and plated for bacterial CFUs (Fig 2G). Together, these data show a strong increase in intracellular *Bc* replication in cells lacking the ability to respond to type I IFN and provide a basis for further exploration into why this is the case.

### Increased cell death in *Bc*-infected *Ifnar1*-/- macrophages does not depend on inflammasome activation

Infection with *Bc* can drive inflammasome activation leading to pyroptotic cell death [35]. Since we observed elevated cell death in *Ifnar1*^-/-^ iBMDMs infected with *Bc* (S2D and S3A Figs), we sought to examine the impact of type I IFN signaling on inflammasome activation and pyroptosis in *Bc*-infected cells compared to inflammasome-deficient *Casp1/11*^*-/-*^ cells. At 24 h post-infection with J2315, WT BMDMs showed pronounced cleavage of both Caspase-1 and Gasdermin-D, which was either absent or reduced in *Casp1/11*^*-/-*^ and *Ifnar1*^-/-^ BMDM, respectively (S3C Fig). Furthermore, release of IL-1β was dampened in *Ifnar1*^-/-^ cells (S3D Fig). Intriguingly, cell permeation measured by Draq7 uptake was slightly elevated in *Ifnar1*^-/-^ BMDMs, while *Casp1/11*^*-/-*^ inflammasome-defective cells showed only a transient lag in Draq7 uptake (S3D Fig). Together, these data are consistent with a role for type-I IFN signaling in priming inflammasome gene expression, but suggest that additional cell death pathways contribute to cell permeation in *Bc*-infected macrophages and that the increased cell death seen in *Ifnar1*^-/-^ macrophages infected with *Bc* is largely independent of inflammasome-mediated pyroptosis.

### Species-specific intracellular bacterial replication is differentially impacted by type I and type II IFNs

**Fig 3:**
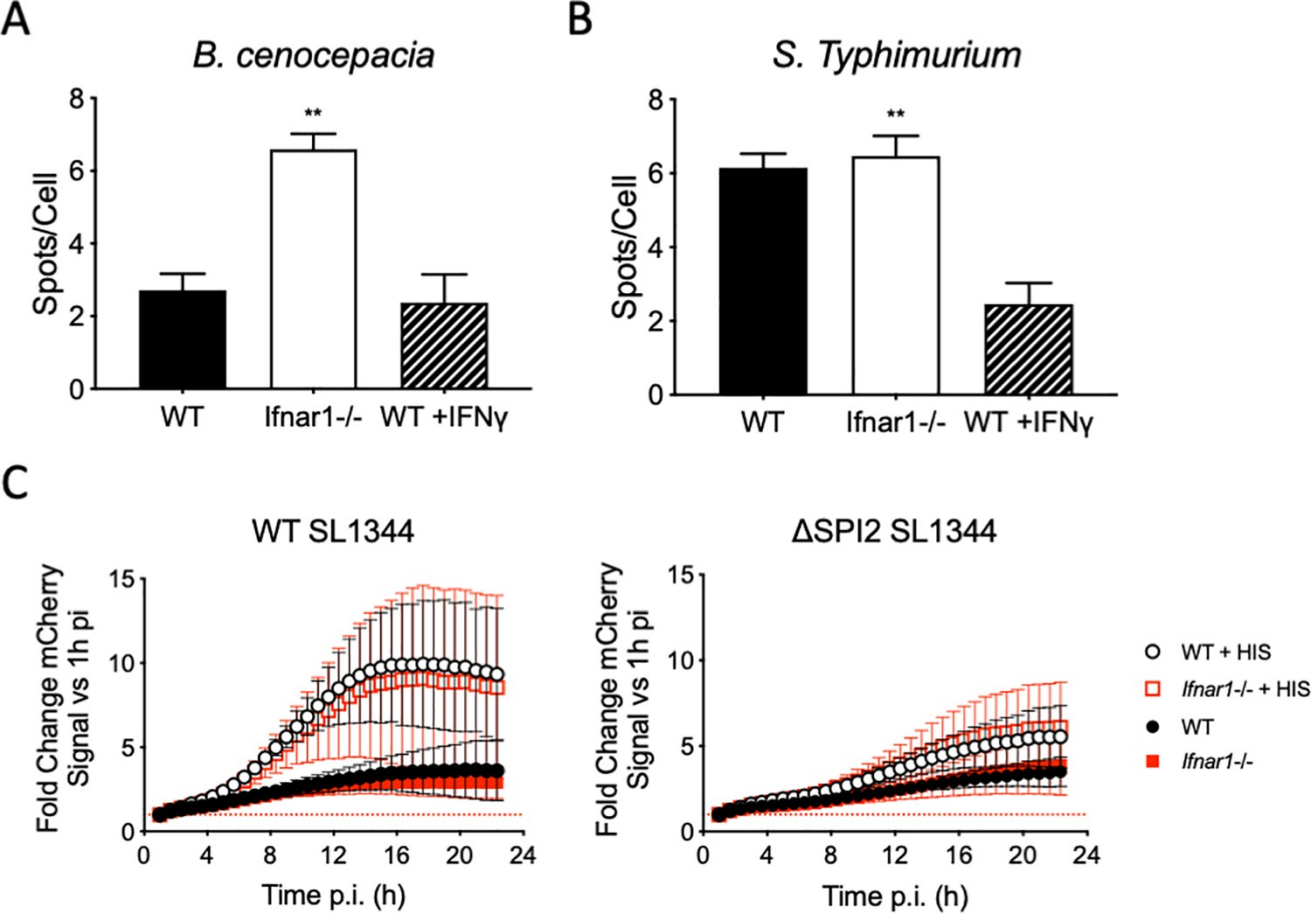
Type I and II IFNs have bacterial species-specific effects on intracellular replication. A,B) WT and *Ifnar1*^-/-^ iBMDMs were stimulated with IFNγ or regular media for 24 h before being infected with A) dsRed+ J2315 (MOI = 1) or B) GFP+ *STm* (MOI = 10) for 22 h. Bacterial levels were measured using high-content imaging. C) BMDMs from WT and *Ifnar1*^-/-^ mice were infected with mCherry WT and ΔSPI2 *STm* strain SL1344 (MOI = 15) for 24 h in the presence and absence of histidine. Total mCherry signal was measured every 20 min and signal normalized to 1 h pi. * = p≤0.05, ** = p≤0.01, and **** = p≤0.0001 by Mann-Whitney U test.

The lack of increase in microbicidal activity against *Bc* in macrophages stimulated with IFNγ was surprising, especially considering the extensive literature showing IFNγ-based bacterial killing in multiple bacterial infection systems [31–33]. As such, we were curious to compare the roles of IFNγ and IFNβ on the replication of other macrophage-invading bacteria, such as *Salmonella enterica* serovar Typhimurium (*STm*) in our infection system. In our infection system, this pathogen is taken up into macrophages via phagocytosis, much like *Bc*, but largely does not escape into the cytoplasm. Instead, *STm* induces changes in the phagosome that repurpose the vacuole into a replicative niche [34]. Unlike *Bc*, intracellular *STm* replication is potently blunted by IFNγ pretreatment, so we cross-compared *STm* against *Bc* replication within *Ifnar1*^-/-^ iBMDMs, to prevent endogenous Type-I IFN autocrine signaling, or pre-stimulating iBMDM with IFNγ, which is only present by addition from an exogenous source, followed by high-content imaging. As expected, *Bc* replication was increased in *Ifnar1*^-/-^ iBMDMs compared to WT cells, and pre-stimulating the cells with IFNγ had no effect on replication (Fig 3A). The direct opposite was true of *STm* replication, with no change in bacterial load when comparing WT and *Ifnar1*^-/-^ iBMDMs and a significant reduction in bacterial replication when WT iBMDMs were pre-stimulated with IFNγ (Fig 3B). Furthermore, *Ifnar1*^-/-^ BMDMs exhibited no loss of restriction of *STm* regardless of whether infection conditions support fast growing bacilli (histidine supplementation) or slow growing bacilli (genetic *Salmonella* pathogenicity island-2 type-III secretion system deletion) during live imaging experiments. (Fig 3C). Surprisingly, although there’s a differential impact on bacterial replication of *Bc* and *STm* following discrete types of IFN reprogramming, deletion of IFNAR led to increased cell death irrespective of whether infections were performed with *Bc* or *STm* (S3A and S3B Fig). These data suggest an interesting dichotomy when it comes to the importance of IFN signaling during intracellular bacterial infection, while also highlighting non-redundant effects of type I versus type II IFN stimulation, which would be an important topic for future investigation.

### IFN signaling has profound effects on macrophage transcriptional responses to LPS and cytosolic *Bc* infection

Type I IFNs act by signaling through a heterodimeric receptor complex made up of IFNα/β receptor 1 (IFNAR1) and IFNAR2 chains. Ligation of IFNAR by IFNs leads to signaling via the JAK/STAT pathway and the subsequent transcription of a class of genes known as IFN-stimulated genes (ISGs), many of which have been implicated in protecting cells from viral infection, halting cell proliferation, and supporting both pro- and anti-inflammatory cytokine production [13]. What constitutes the ISG family is a source of great debate in the immunology community, as IFNAR signaling can have profoundly disparate effects depending on cell type, stimulus, microenvironmental changes, and the method by which transcription is measured. A short list of two hundred or so ’prototypical’ ISGs including *IFITs*, *RSADs*, and various chemokines are often used as proxies for interferon-based gene readouts, though greater than 4000 genes have been shown to be regulated, in some way, by IFN stimulation [36–38]. By stimulating WT and *Ifnar1*^-/-^ BMDMs with either J2315 or Kdo2-Lipid A (KLA, the immune-stimulatory portion of LPS) and collecting RNA at various time points for RNA sequencing, we aimed to better understand the nature of the IFN-dependent transcriptional response to cytosolic Gram-negative bacterial infection and, especially, how it may differ from the LPS response.

**Fig 4:**
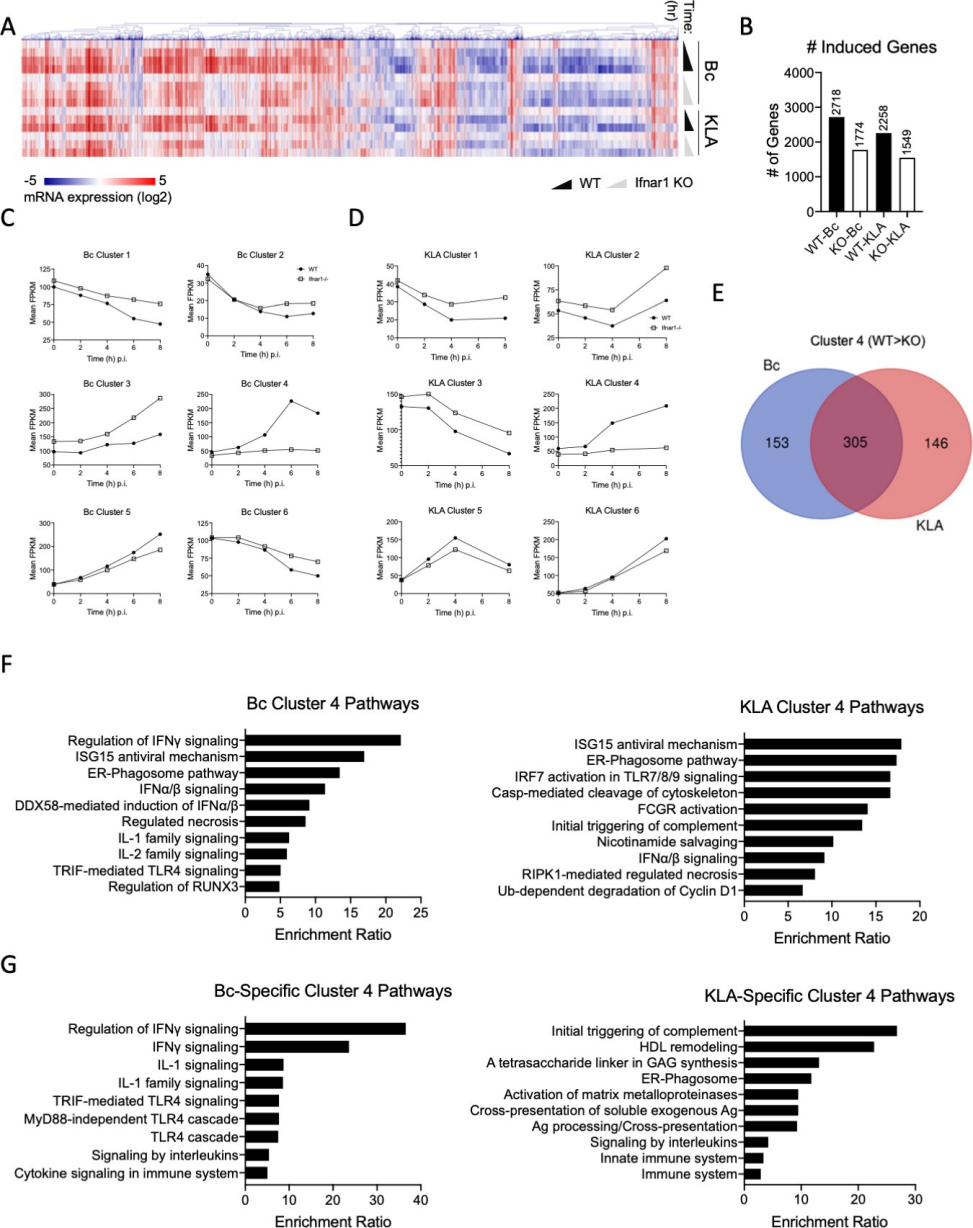
IFN signaling has profound effects on macrophage transcriptional responses to LPS and cytosolic bacterial infection. WT and *Ifnar1*^-/-^ BMDMs were infected with wt *Bc* (MOI = 1) or stimulated with KLA (5nM) for the given amounts of time before RNA was extracted and sequenced. A) Heat map showing relative gene expression across times 0, 2, 4, 6, and 8 h post-stimulation. Each column is a gene with descending rows representing elapsed time. Color scale runs from blue to red, representing log_2_ fold-change. Genes included reached log_2_ FC > 1 or < -1 with FDR < 0.05 in *at least* 3 conditions, leaving us with 3918 genes. B) Number of genes that reach log_2_ fold-change > 1, FDR ≤ 0.05 across the time course for each genotype and stimulation. C,D) *maSigPro* output of gene clusters based on the ‘shape’ of expression dynamics across the time course for cells infected with *Bc* (C) or stimulated with KLA (D). Only genes where log_2_ fold-change > |1|, FDR ≤ 0.05 were included in clusters. E) Venn diagram showing the overlap between Cluster 4 gene members in *Bc*-infected and KLA-stimulated cells. F) Top 10 pathways associated with each of the Cluster 4 gene members, as found by Reactome pathway analysis. G) Top 9 pathways associated with the Cluster 4 genes *specific* to each stimulation, as found by Reactome pathway analysis. N = 3 for each stimulation condition.

Firstly, we performed a simple paired differential expression analysis for all groups relative to the time 0 h group (e.g., 2 h KLA stimulation vs 0 h, 4 h *Bc* stimulation vs 0 h, etc.), looking at how many genes were upregulated at each timepoint of stimulation (Figs 4A, 4B and S4A). In WT cells, across the timecourse, 2718 genes were upregulated upon infection with *Bc*, while 2258 genes were induced during KLA stimulation (log_2_ fold-change > 1, FDR ≤ 0.05). Conversely, 1774 genes were upregulated in *Ifnar1*^-/-^ BMDMs when infected with *Bc* while 1549 genes increased during KLA stimulation. For each stimulus, the number of genes upregulated increased over the course of the 8 hours, with *Bc* infection having a greater effect later in the timecourse than KLA stimulation (S4A Fig). We then counted how many genes were being transcribed at a higher level in WT cells than in the KO cells *at any point in the timecourse* by isolating genes that had fold-change values at least twice as high in WT cells than KO cells at each timepoint. Through this analysis we found 1422 genes in KLA-stimulated populations and 1934 genes in the *Bc*-infected cells (S4B Fig). This suggests a substantially greater type I IFN dependence for the macrophage transcriptional response to bacterial challenge than previously appreciated. While the transcription of all of these genes may not entirely depend on IFN stimulation, IFN has a substantial role in supporting their expression during both bacterial infection and KLA stimulation.

We next used the R package *maSigPro* [39] to test for significantly different expression between the KO and WT through time for each treatment group. All statistically significant genes (FDR-corrected *p*-value ≤ 0.05, R^2 >^ 0.7) were clustered into six distinct temporally variable clusters for each WT vs *Ifnar1*^-/-^ comparison (Fig 4C and 4D). For both stimuli, cluster 4 represents what we would consider ’true ISGs’. These are genes that are significantly upregulated in WT cells, but not *Ifnar1*^-/-^ cells. When we compared the genes in cluster 4 for each stimulus, we found that approximately half (305 out of 604 total genes across both stimuli) were shared, while 153 and 146 genes were restricted to the Bc or KLA group, respectively (Fig 4E). Pathway analysis of both Cluster 4s showed many expected significantly enriched pathways (FDR < 0.05), with both Bc and KLA showing enrichment for Type I IFN signaling pathways (e.g., ‘IFNα/β signaling’) as well as additional innate immune system pathways and the ER-phagosome pathway (Fig 4F). When examining pathways enriched for genes found only in the *Bc*-infected cells, the top enriched pathways were related to IFNγ signaling, while KLA-specific genes were enriched for the complement system and antigen cross-presentation (Fig 4G). Interestingly, Cluster 3 of the *Bc* dataset (Fig 4C), contains genes that were significantly more highly upregulated in the *Ifnar1*^-/-^ macrophages. This cluster does not exist in the KLA dataset. Pathway analysis showed that many of these genes are associated with metabolic pathways, including ‘Metabolism’, ‘Detoxification of ROS’, and ’Glutathione Conjugation’ (S4C Fig). However, simple metabolic assays did not show a significant difference between J2315-infected WT and *Ifnar1*^-/-^ iBMDMs. Using a Seahorse XF acidification assay, we found no significant difference in the shift to glycolysis between WT and *Ifnar1*^-/-^ iBMDMs infected with *Bc*, as measured by increased proton efflux rate (S5A Fig). We also measured reactive oxygen species (ROS) production in infected WT and Ifnar1^-/-^ cells. While we did see increased basal H2O2 levels in *Ifnar1*^-/-^ iBMDMs compared to WT, and a significant reduction in H2O2 after infection with a high dose of *Bc* (S5B Fig), these infection-based differences disappeared when H2O2 levels were normalized to uninfected cells (S5C Fig). We therefore chose to focus our follow-up efforts on gene members of Cluster 4.

### ISGs play a critical role in the transition to autophagy during intracellular bacterial infection

**Fig 5:**
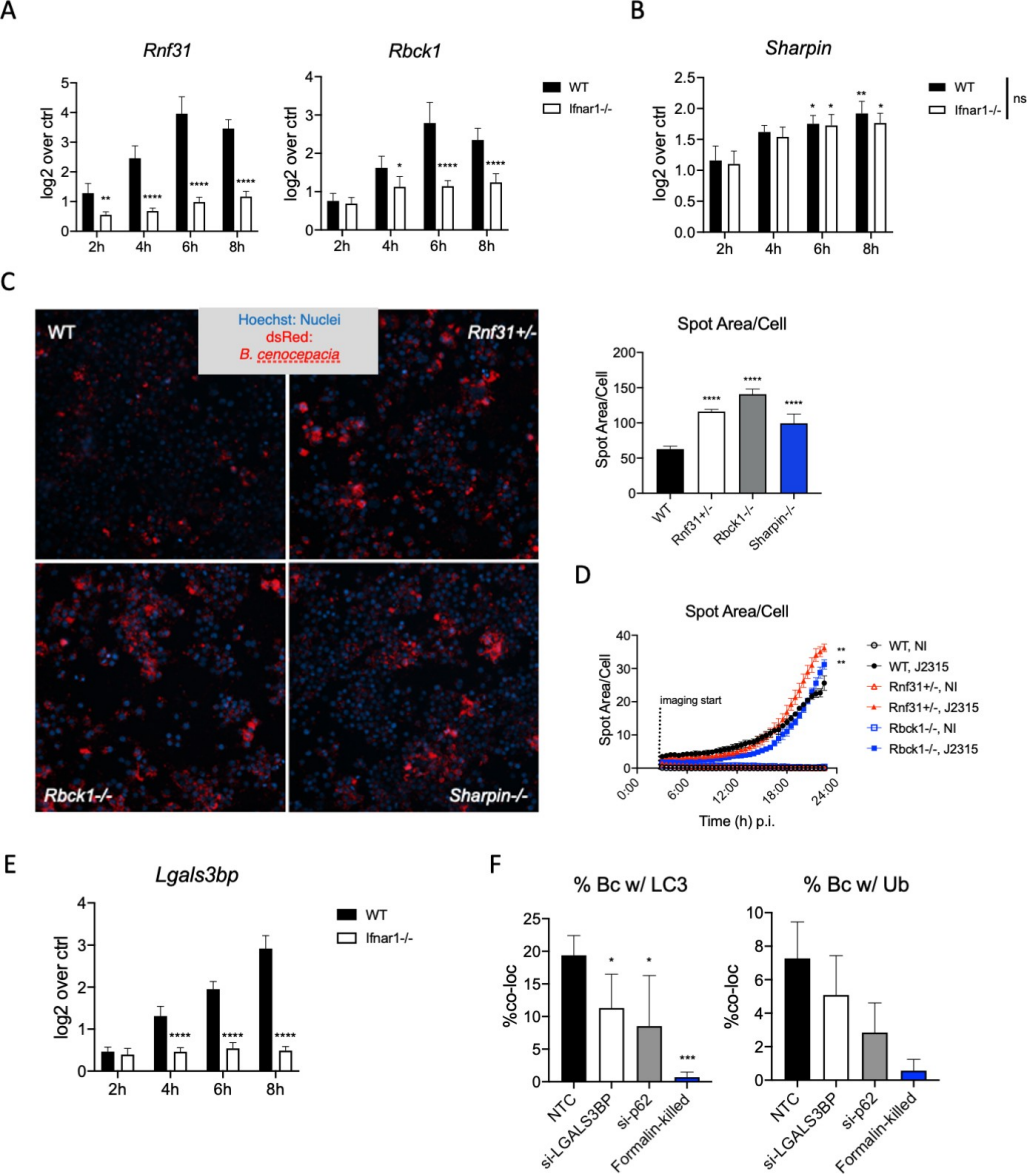
ISGs play a critical role in the transition to autophagy during intracellular bacterial infection. A,B) WT and *Ifnar1*^-/-^ iBMDMs were infected with wt *Bc* (MOI = 1) for given amounts of time before RNA was extracted. A) *Rnf31*, *Rbck1*, or B) *Sharpin* transcription was measured by RT-PCR and normalized to *Hprt* in uninfected cells. n = 5 for each timepoint, representative of 2 independent experiments. C) WT, *Rnf31*^+/-^, *Rbck1*^-/-^, and *Sharpin*^-/-^ RAW264.7 cells were infected with dsRed+ *Bc* (MOI = 1) for 22 h before fixing with PFA. Bacterial fluorescence was analyzed by high-content imaging using dsRed fluorescence. D) WT, *Rnf31*^+/-^, and *Rbck1*^-/-^ RAW264.7 cells were infected with dsRed+ *Bc* (MOI = 1) and bacterial fluorescence was measured every 30 min for 22 h by live-cell high-content imaging. Infection experiments have n = 5 and are representative of 3 independent experiments. E) WT and *Ifnar1*^-/-^ iBMDMs were infected with wt *Bc* (MOI = 1) for given amounts of time before RNA was extracted. *Lgals3bp* transcription was measured by RT-PCR and normalized to *Hprt*. n = 5, representative of 2 independent experiments. F) PMA-differentiated THP-1 cells were infected with *Bc* for 6h before fixing and staining for LC3B and total ubiquitin. Co-localization of *Bc* with LC3 or Ub were enumerated by high-content imaging followed by spot detection analysis. Co-localization experiments were done with n = 5, and are representative of 2 independent experiments. * = p≤0.05, ** = p≤0.01, and **** = p≤0.0001 by Mann-Whitney U test. Comparisons in 5B are between individual groups with their respective unstimulated cells (i.e. WT vs. WT, KO vs. KO). There were no significant differences between WT and KO responses for this assay.

Two genes sorted into Cluster 4 for both *Bc* infection and KLA stimulation were notable for their putative roles in the host autophagy response to bacterial infection. We have shown previously that autophagy is vital for anti-*Burkholderia* immunity, and that boosting the autophagic response can successfully protect macrophages from *Bc*-induced cell death [12]. The first gene of interest was *Rnf31*, which encodes the protein HOIP, a central member of the linear ubiquitin chain assembly complex (LUBAC). The LUBAC complex has been previously linked to antimicrobial responses in the cytosol, with the deposition of linear ubiquitin chains on the surface of bacteria being a key step in recruiting optineurin and initiating the autophagy response [40]. We collected RNA from J2315-infected iBMDMs early in infection and ran RT-PCR for both *Rnf31* and *Rbck1* (also known as HOIL-1, another key member of LUBAC) and found that both were transcribed at significantly greater levels in WT cells compared to *Ifnar1*^-/-^ cells, supporting the difference found in the RNAseq dataset (Fig 5A). *Rbck1* did not reach significance in the original RNAseq experiment, but did when using quantitative PCR. The third core member of LUBAC, *Sharpin*, was not significantly affected by the absence of IFNAR on macrophages, though it was upregulated during *Bc* infection (Fig 5B). We hypothesized that decreased expression of LUBAC components would lead to decreased ubiquitination of cytosolic bacteria and increased bacterial replication. We therefore generated mouse macrophage RAW264.7 cell lines with perturbed expression of LUBAC components using CRISPR/Cas9-based gene editing. Indeed, when we infected cells with dsRed-expressing J2315 for 22h, we saw increased bacterial burden in *Rnf31*^+/-^, *Rbck1*^-/-^, and *Sharpin*^-/-^ cells compared to WT cells (Figs 5C and S6A). Using live-cell imaging, we found that differences in bacterial replication only became apparent towards the end of the infection (Fig 5D and S1 Movie). These data suggest that type I IFN signaling leads to an increase in LUBAC components, which are key drivers of the later phase anti-*Bc* immunity when bacteria escape to the host cell cytosol. However, when cells deficient in LUBAC components were pre-treated with exogenous IFNβ and then infected with *Bc*, they did show a decrease in bacterial abundance by live-cell imaging (S6B Fig). This is likely due to multiple IFN-stimulated pathways contributing to anti-*Bc* immunity in macrophages and will require further investigation to uncover completely.

Another gene of interest from Cluster 4 was *Lgals3bp*, which encodes a Galectin-3 binding protein. The majority of LGALS3BP protein produced by cells is heavily glycosylated, then secreted by cells so it can interact with the extracellular matrix. However, recent findings have highlighted important roles for this protein as an IFN-induced signaling scaffold during viral infection [41], and it has been shown to associate with certain bacterial proteins within infected cells [42]. To determine whether LGALS3BP plays a role in anti-*Bc* responses, we first measured *Lgals3bp* transcription in *Bc*-infected WT and *Ifnar1*^-/-^ iBMDMs to ensure our RNAseq data was accurate. Indeed, *Lgals3bp* transcription was heavily attenuated in *Ifnar1*^-/-^ macrophages compared to their WT counterparts, with the timing of transcription following a similar pattern to the other ISGs we have measured in this study (Fig 5E). In order to test the importance of *Lgals3bp* during *Bc* infection, we reduced *Lgals3bp* expression in differentiated THP-1 macrophages via siRNA knock-down (S6C Fig) and measured LC3B and Ub co-localization with the bacteria to determine whether LGALS3BP plays a role in the initiation of autophagy. Reducing levels of LGALS3BP in THP-1 cells led to decreased LC3B co-localization with J2315 with similar levels to when we knocked down p62, a critical regulator of autophagy (Fig 5F). We also saw decreased Ub co-localization in si-LGALS3BP cells (Fig 5F). Altogether, these data suggest important roles for ISGs in both the LUBAC complex and LGALS3BP in coordinating the autophagic response to *Bc* in the cytosol, providing a strengthened link between type I IFN signaling and the cell-autonomous antibacterial response.

### IFN-mediated protection in macrophages relies on prolonged IFN production through cytosolic PRRs

**Fig 6:**
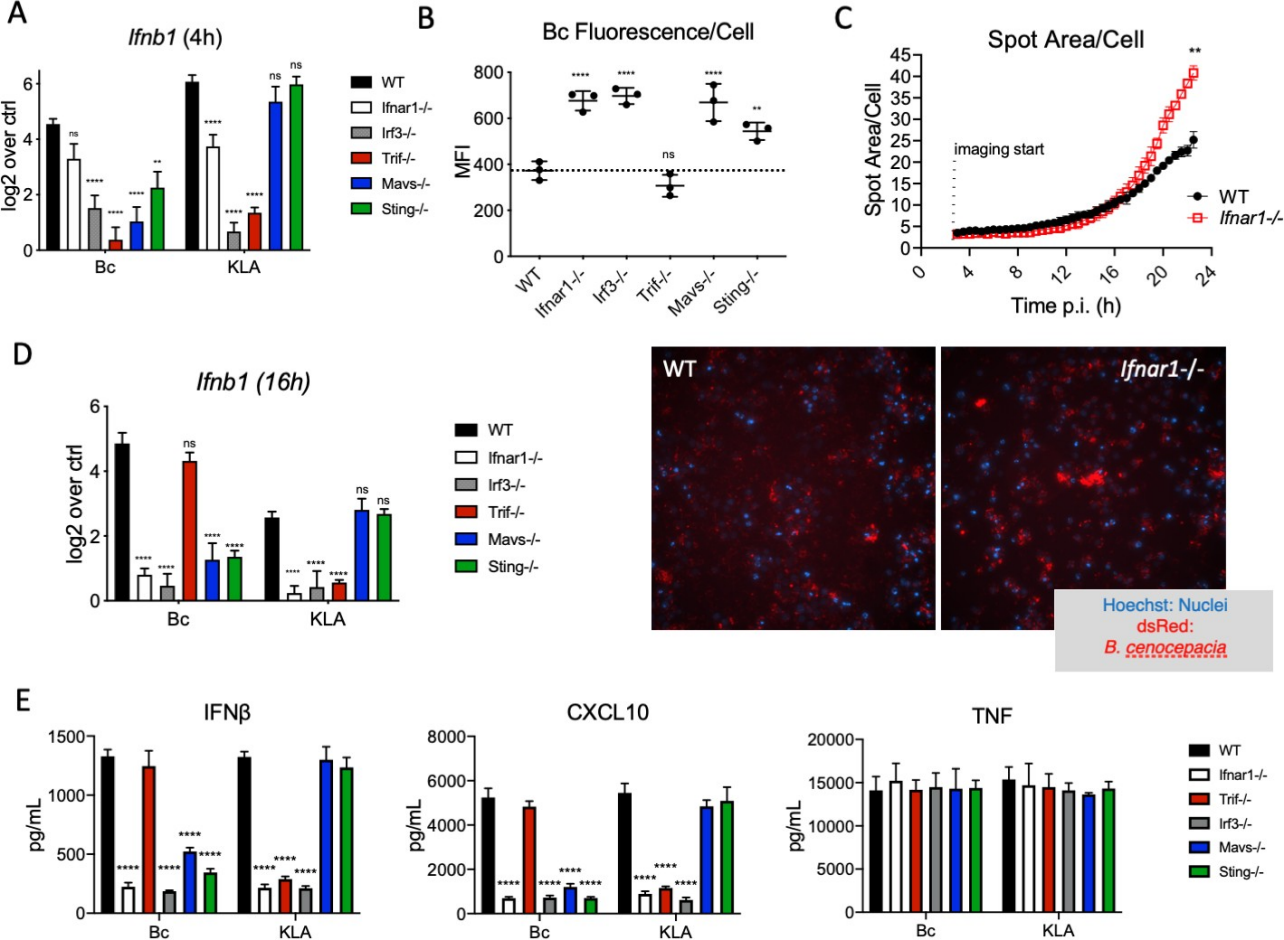
IFN-mediated macrophage protection relies on prolonged IFN production through cytosolic PRRs. A) The indicated IMM cell lines were infected with live *Bc* (MOI = 1) or stimulated with KLA (5nM) for 4 h. *Ifnb1* transcription was measured by RT-PCR and normalized to *Hprt*. B) The same IMM lines were infected with live dsRed+ *Bc* (MOI = 1) for 22 h and bacterial load was measured via flow cytometry. C) [includes imaging panels below] WT and *Ifnar1*^-/-^ iBMDMs were infected with dsRed+ *Bc* (MOI = 1) and bacterial growth was measured using live-cell high-content imaging based on dsRed fluorescence. D, E) IMM lines were infected with either live *Bc* (MOI = 1) or stimulated with KLA (5 nM) for 16 h (D) or 24 h (E). D) *Ifnb1* transcription was measured by RT-PCR and normalized to *Hprt*. E) Cell culture supernatants were analyzed for cytokine concentration by LegendPLEX assay. All experiments were performed with n ≥ 3, and are shown as representative of ≥ 3 independent experiments. ** = p≤0.01 and **** = p≤0.0001 by Mann-Whitney U test.

Since many of the ISGs activated during *Bc* infection showed strong induction across an extended time course, we sought to determine which IFN-inducing PRR pathways were responsible for driving the host-protective ISG program. The production of IFNs by macrophages upon stimulation with Gram-negative bacteria like *Bc* is often associated with TLR4 signaling from the endosome [19]. More specifically, the endocytosis of bacteria into TLR4-containing endosomes leads to the activation of the adaptor protein TRIF, leading to the eventual activation of IRF3 and the transcription of type I IFNs and other IFN-associated genes. Because *Bc* is actively engulfed by macrophages, we hypothesized that TLR4 signaling through TRIF would be the primary source of signaling leading to IFN transcription, and this was supported by pathway enrichment of *Bc* cluster 4 genes (Fig 4F) where TRIF-mediated TLR4 signaling was significantly enriched. To test this, we infected WT, *Ifnar1*^*-/-*^, and *Trif*^*-/-*^ iBMDMs with live J2315 or stimulated them with KLA for 4 hours before extracting mRNA and performing RT-PCR for the *Ifnb1* gene. At this time point, *Trif*^-/-^ iBMDMs were transcribing significantly less *Ifnb1* than WT or *Ifnar1*^-/-^ iBMDMs, and similar amounts as *Irf3*^-/-^ cells, which we used as a negative control of *Ifnb1* transcription (Fig 6A). This was true of both *Bc* infection and KLA stimulation, supporting our hypothesis that IFNβ production occurs downstream of TRIF signaling during *Bc* infection of macrophages. However, when we infected *Trif*^-/-^ iBMDMs with dsRed+ J2315 and measured bacterial replication by flow cytometry, there was no significant difference between these cells and WT iBMDMs, despite the expected increase in bacterial load in *Ifnar1*^-/-^ and *Irf3*^-/-^ cells (Fig 6B). Therefore, TRIF-dependent IFN production was not necessary for protecting macrophages from the increased abundance of *Bc* found in *Ifnar1*^-/-^ cells.

Alternative pathways leading to IFN production downstream of bacterial infection include those linked to the cytosolic nucleic acid receptors MAVS and STING [43–45]. We hypothesized that these receptors were contributing to the production of IFNβ during *Bc* infection and that these were perhaps able to compensate for the lack of TRIF-dependent signaling in the previous experiments. Indeed, when we infected *Mavs*^-/-^ or *Sting*^-/-^ iBMDMs with *Bc* for 4 hours, we also saw a reduction in *Ifnb1* transcription compared to WT cells (Fig 6A). Importantly, we did not see this effect when stimulating these iBMDMs with KLA, as these receptors are unable to recognize cytosolic TLR4 ligands. Similarly, multiple TLR4 pathways are significantly enriched in the *Bc*-specific cluster 4 genes but were completely absent from the KLA stimulation (Fig 4F). In contrast to the lack of a *Bc* replication effect in *Trif*^-/-^ iBMDMs, we observed a significant increase in replication in *Mavs*^-/-^ or *Sting*^-/-^ iBMDMs compared to WT cells, and this increase was similar to *Ifnar1*^-/-^ cells (Fig 6B). These data caused us to reconsider our assumed model of type I IFN production during *Bc* infection to one that involved a more prolonged IFN response downstream of these cytosolic sensors. Indeed, when we performed a timecourse of bacterial replication in WT and *Ifnar1*^-/-^ iBMDMs, we saw that the increased bacterial abundance phenotype we’d seen in *Ifnar1*^-/-^ cells only emerged late in the infection (Figs 2G and 6C and S2 Movie). Even 16 hours post-infection there was no significant difference between the replication in either cell population. This led us to measuring *Ifnb1* transcription at 16 h post-infection (or post-stimulation with KLA) in various IMM cell lines to determine the relative importance of TRIF, MAVS, and STING to the production of IFNβ at this critical stage following bacterial challenge (Fig 6D). In *Bc*-infected cells, *Ifnb1* transcription was significantly reduced when those cells lacked any of IFNAR1, IRF3, MAVS, or STING expression, but *Trif*^-/-^ iBMDMs showed normal *Ifnb1* transcription at this later time point. In KLA-treated cells, however, MAVS and STING were expendable for *Ifnb1* transcription, while TRIF was required, as expected. These receptor dependencies for *Ifnb1* transcription also extended to protein production, as IFNβ protein was found in supernatants of *Trif*^-/-^ iBMDMs infected with *Bc* for 24h, but not those from cells lacking MAVS or STING (Fig 6E). Conversely, KLA-stimulated cells did not produce IFNβ when the cells lacked TRIF, but those lacking STING or MAVS produced normal amounts of the cytokine. We also measured protein levels of CXCL10, also known as IFNγ-induced protein 10 (IP-10), which was found at significantly higher levels in the supernatants of WT and *Trif*^-/-^ iBMDMs infected with *Bc* than those of *Ifnar1*^-/-^, *Irf3*^-/-^, *Sting*^-/-^, or *Mavs*^-/-^ iBMDMs, with no difference in TNF production (Fig 6E).

Finally, we tested whether the TRIF, MAVS and STING sensory pathways impacted traditional LC3B autophagosome formation via high-content imaging, since this pathway is known to be important in anti-*Bc* immunity. We observed two waves of LC3B activation (S7A Fig), likely correlating with initial cytosolic escape of *Bc* 2–4 hr after infection, and then again during increased cytosolic replication at 16–20 hr [11,12]. Furthermore, *Trif* ^-/-^ iBMDM exhibit partially diminished autophagy responses at both phases, while the later phase response was almost completed ablated in *Sting*^-/-^ cells (S7A and S7B Fig). While this reduced late LC3B activation may contribute to the increased bacterial replication in *Sting*^-/-^ cells, it is noteworthy that no diminished LC3B response was observed in *Mavs*^*-/-*^ cells, suggesting that the increased *Bc* permissiveness in these cells is less autophagy dependent.

**Fig 7:**
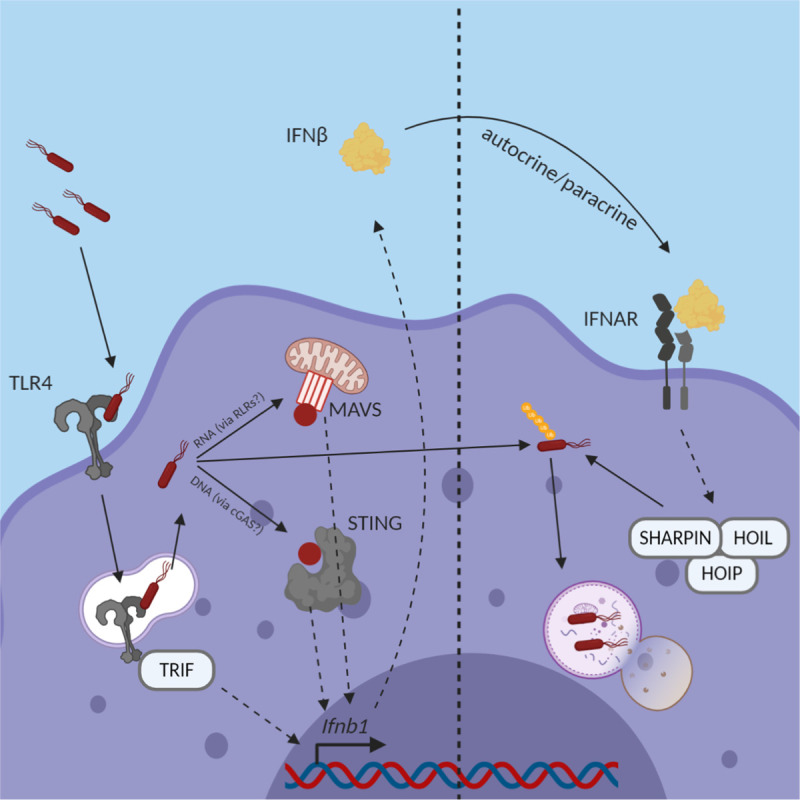
*Schematic of* Bc-*induced IFN production and effects*. When *Bc* enters the lungs, it is recognized by alveolar macrophages and taken up by these phagocytes. TLR4 sensing in the endosome leads to *Ifnb1* transcription via the TRIF/TRAM pathway. However, bacterial escape to the cytosol allows for the integration of other signaling pathways like cGAS-STING and the RLR-MAVS pathway, which also contribute to *Ifnb1* transcription, and allow for prolonged IFNβ production. IFNβ acts in an autocrine/paracrine fashion by binding to its receptor, IFNAR, leading to the transcription of various ISGs, including components of the LUBAC complex such as HOIP, HOIL-1, and SHARPIN. The LUBAC proteins then tag the cytosol-dwelling *Bc* for destruction via the autophagy pathway.

Together, these data suggest that the initial recognition of *Bc* via the TLR4/TRIF pathway supports early IFNβ production, but not the IFN and ISG-dependent reduction in bacterial replication. Instead, sustained sensing of cytosolic *Bc* via pathways involving MAVS and STING provides the persistent IFN production necessary for limiting bacterial replication in macrophages. This sustained IFN signaling leads, in part, to increases in the autophagy machinery necessary to engulf and destroy cytosol-dwelling *Bc*, allowing for the macrophage to expediently deal with acute opportunistic infections without the need for adaptive immune type II IFN responses (model outlined in Fig 7).

## Discussion

Since their discovery, it has been clear that type I IFNs are an integral part of the antiviral innate immune response. Indeed, these cytokines were first described due to their ability to reduce the replication rate of influenza viruses. In the ensuing decades, it has become clear that IFNs have important roles in a remarkable number of immune and homeostatic mechanisms, including cell proliferation, inflammation, antigen presentation, immunity to countless pathogens, and metabolism, among others. However, despite their pleiotropic nature, the vast majority of IFN-related research still focuses on their antiviral mechanisms. Type I IFNs are also important components of the antibacterial response, though they are not always protective to the host [46]. Many bacterial pathogens have evolved in order to subvert IFN-based immunity or else skew it to promote IFN’s anti-inflammatory effects. For example, type I IFN signaling enhances *Listeria monocytogenes* pathogenesis due to an increase in apoptosis [47] and attenuation of IFNγ and IL-17 responses [48,49]. Another common example is secondary bacterial infections wherein a strong IFN response to a virus such as influenza leads to aberrant anti-bacterial responses, especially towards *Streptococcus pneumoniae* [50–54]. Finally, perhaps the best-described interaction between type I IFNs and a bacterial infection is that of *Mycobacterium tuberculosis* (*Mtb*). Patients with active tuberculosis have a pronounced ISG signature in their blood cells [55], and *Ifnar1*^-/-^ mice are better equipped to clear *Mtb* from the lungs, though this depends heavily on both the strains of mice and bacteria used [56,57]. WT mice are also more susceptible to *Mtb* if co-infected with influenza due to elevated type I IFN production [57].

Despite the above examples of type I IFNs being detrimental to the host during bacterial infection, this is not always the case. For example, WT mice infected with *Streptococcus pyogenes* showed significantly greater survival than those lacking either IFNAR1 or IFNβ [58]. Similarly, there is evidence that *Legionella pneumophila* replication in macrophages is diminished through type I IFN signaling [59], *Rickettsia parkeri* relies on inflammasome-mediated cell death to escape host-protective type I IFN responses and allow for bacterial growth [60] and type I IFN supports resolution of peritonitis and pneumonia-induced inflammation [61]. While not an exhaustive list, these studies show that the role of type I IFNs in bacterial infections is complex, especially at the level of the organism, and more work needs to be done to describe the many relationships among these cytokines, immune cells, and the bacteria that infect them.

In contrast to the bacteria described above, *Bc* is not a ‘professional’ pathogen in mammals and only causes disease in individuals with underlying immunodeficiencies, such as cystic fibrosis, chronic granulomatous disease, and those on immunosuppressive drugs [8]. Indeed, even naïve WT mice that are infected via the lungs with large doses of *Bc* show no outward signs of infection (Fig 1D). As such, we hypothesized that basic innate immune responses including IFN production, are sufficient to eliminate this infection in immunocompetent individuals. This is important not just in the context of *Bc* specifically, but likely contributes to our immune system’s ability to clear a multitude of environmental pathogens. The importance of this cannot be overstated as many of these microbial species, and especially those that live in the soil like *Bc*, are often the most resistant bacterial species to our current antibiotics. Because these bacteria live in complex and ever-changing communities, the most successful species are those that are able to fight off other micro-organisms through antimicrobial resistance. Protecting the body from these potential pathogens is an important role of the innate immune system and, as we show here, type I IFNs specifically.

One interesting aspect of the anti-*Bc* macrophage response is the inability of exogenous IFNγ to reduce bacterial replication in type I IFN-competent WT cells. While the downstream transcriptional effects of type I and type II IFNs overlap significantly, they are not the same [62]. However, the autophagy-related genes we identified later in this study are, indeed, positively regulated by both type I and type II IFNs [63]. Therefore, there are likely other important type I IFN-specific mediators that contribute to antimicrobial immunity in macrophages. IFNγ is predominantly produced during the adaptive immune response, especially by CD4+ helper T cells. Because macrophages are unable to produce their own IFNγ, it is important in this context that they are able to boost cell-autonomous antibacterial responses without the need for exogenous stimulation. Macrophages produce significant amounts of IFNβ, making them ‘self-sufficient’ in terms of responding to sub-pathogenic threats such as *Bc* infection. This is highlighted in our *in vivo* infection studies as well, where the loss of type I IFN signaling led to significantly more weight loss than during infection of WT mice.

IFN production is induced in macrophages downstream of endosomal TLR4 signaling during both bacterial infection and purified LPS stimulation [19,64,65]. Despite this commonality, the transcriptional programs induced by IFNs are not identical in these two situations. As we show in Fig 5, the size and structure of the IFNAR-dependent transcriptional response varies based on the stimulus, even early on. The most striking of these differences is the inclusion of a cluster of genes in the *Bc*-infected cells whose transcription is repressed by IFNAR signaling but are not induced in *Ifnar1*^-/-^ cells stimulated with KLA. Pathway analysis of this gene set uncovered metabolic pathways and oxidative stress responses, implicating IFN signaling in metabolic reprogramming of these macrophages–something that has been shown in other cell types, and especially during viral infection [66,67]. However, we were unable to find any significant differences in ROS dynamics or extracellular acidification between *Bc*-infected WT and *Ifnar1*^-/-^ macrophages.

The discovery that endosomal TLR4 signaling links bacterial recognition to type I IFN production solidified the evolutionary importance of these cytokines for our protection against potentially pathogenic bacteria [19,24]. Since then, the number of PRRs that link bacterial infection with IFN production has grown to include many cytosolic receptors including MAVS and STING. These receptors recognize nucleic acid products, including those generated from bacterial DNA in the cytosol. STING is activated by cyclic dinucleotides that are either produced by bacteria or else generated by the host enzyme cGAS [68]. It is known that *Bc* produces cyclic dinucleotides through its own cGAS-like enzyme, though this is the first evidence that recognition of these contributes to anti-*Bc* immunity in macrophages [68]. Likewise, activation of MAVS has been shown to occur during intracellular bacterial infection, likely through the recognition of bacterial mRNA by RIG-I [44]. Both of these receptors have been linked to recognition of both pathogenic and non-pathogenic bacteria [43,44]. This again points to the importance of IFN regulation in the context of infection with non-professional pathogens like *Bc*, which can be cleared efficiently so long as type I IFN signaling remains intact. The cytosolic lifestyle of these bacteria also provides ample opportunity for recognition by these nucleic acid-sensing pathways, leading to the sustained IFN responses we show to be so important for host defense. This is in contrast to endosomal TLRs, whose signaling cannot be triggered from the cytosol. This sustained sensing and signaling could contribute to the differences we see in the transcriptomes of cells infected with *Bc* compared to KLA stimulation.

We have previously shown that the autophagic capacity of macrophages plays a vital role in eliminating *Bc* infection in healthy individuals while primary immunodeficiencies that limit autophagy, such as CF and CGD, lead to uncontrolled *Bc* infection which is often lethal [12,69]. Autophagy mediators like LC3B and closely associated anti-microbial effectors are recruited to bacteria soon after their escape from the phagosome, limiting their replication and therefore spread throughout the host. Previous studies have shown that interplay between Type-I IFN signaling and autophagy can potentiate the antimicrobial capacity of cells as a whole, especially in the context of viral infection [70,71]. We have shown here that this extends to intracellular bacterial infection, with IFNAR signaling leading to the upregulation of genes important for enhancing a specific recruitment of selective autophagy, namely through *Rnf31* (HOIP), *Rbck1* (HOIL), and *Lgals3bp*. HOIP and HOIL are members of the LUBAC complex and, as such, play a vital role in marking cytosolic pathogens for removal by autophagy by depositing linear chains of ubiquitin on the surface of bacteria [40]. Together with the association of the specific TetraUb autophagy adapter protein OPTN, a non-canonical GABARAP mediated autophagosome is enlisted to bacteria marked in this manner [72]. This unusual ubiquitination is critical to establish one of many forms of selective autophagy that differentially lead to the removal of diverse cytosolic bacteria.

Compared to the LUBAC complex, the role of LGALS3BP in cellular immunity is less straightforward. There is currently little known about how intracellular levels of LGALS3BP are regulated, other than it being an ISG [41], or what its primary function is. Structural analysis shows a scavenger receptor cysteine-rich (SRCR) domain [42], which could act as a binding domain for bacteria, as it does in the class A scavenger receptor MARCO [73]. Potential roles for LGALS3BP include tethering bacteria to ruptured vacuoles through the dual binding of bacteria and other galectins, or else blocking the actions of Galectin-3, which has been shown to suppress autophagic responses to intracellular *Listeria* [74]. Regardless, further studies on how LGALS3BP interacts with cytosolic bacteria, and how these interactions support cellular immunity, are needed.

Overall, we have shown that the type I IFN response is vital for the protection of macrophages from rampant cytosolic *Bc* replication. IFN-competent mice are unaffected by high-dose *Bc* infection, while those lacking one chain of the type I IFN receptor show significant signs of illness and support increased bacterial replication within alveolar macrophages. Exogenous IFNγ stimulation is unable to improve cellular outcomes in WT macrophages infected with *Bc*, whereas exogenous IFNβ decreases bacterial replication even further. IFN production occurs downstream of TLR4/TRIF signaling, but the beneficial effects of IFN require sustained signaling through cytosolic nucleic acid sensors. IFN signaling in infected macrophages contributes to bacterial killing by stimulating the transcription of genes encoding various autophagic proteins. Overall, sustained signaling through the type I IFN receptor supports an anti-replicative state for *Bc* in macrophages, highlighting the therapeutic potential for type I IFNs in opportunistic infections not easily combatted via antibiotics.

## Materials & methods

### Ethics statement

All animal studies were carried out following the recommendations in the Guide for the Care and Use of Laboratory Animals, 8th Edition (National Research Council) and were approved by the NIAID Animal Care and Use Committee (NIH, Bethesda, MD); Protocol number LISB-4E. Animals were euthanized either before the development of clinical disease or at the defined humane endpoint (development of clinical disease: ruffled fur, hunched posture, lethargy).

### Mice and cell lines

Mice were maintained in specific-pathogen-free conditions and all procedures were approved by the NIAID Animal Care and Use Committee (National Institutes of Health, Bethesda, MD). Wild-type C57BL/6J (stock # 000664) and B6.129S2-Ifnar1^tm1Agt^/Mmjax (*Ifnar1*^-/-^, stock #32045-JAX) were originally purchased from The Jackson Laboratory and subsequently bred in-house as heterozygous pairs. Genotyping of offspring was performed by Transnetyx Inc. using genetic information from The Jackson Laboratory. Bone marrow progenitors isolated from sex- and age-matched mice were differentiated into BMDM during a 6 day culture in complete Dulbecco’s modified Eagle’s medium (DMEM + 10% FBS, 100 U/ml penicillin, 100 U/ml streptomycin, 2 mM L-glutamine, 20 mM HEPES) supplemented with 60 ng/ml recombinant mouse M-CSF (R&D Systems). One day prior to infection or stimulation, cells were rinsed with cold PBS, then scraped from plates using a cell lifter. Cells were then plated in the appropriate tissue-culture-treated plate in complete DMEM and allowed to rest overnight at 37°C, 5% CO_2_, 95% relative humidity prior to stimulation.

To create LUBAC KO cell lines, the appropriate gRNA sequences were cloned into the pSpCas9(BB)-2A-GFP vector (PX458, addgene plasmid #48138). Plasmids were sequenced to confirm proper insertion, then extracted with the Qiagen EndoFree plasmid maxi prep kit. 5 μg of each plasmid were electroporated into 2 million RAW264.7 cells with Lonza nucleofection kit V according to manufacturer’s instructions. Singlet GFP positive cells were sorted into 96-well plates at 17–24 h post-electroporation. Individual cell colonies were picked at 10–14 days after sorting and the sequences were confirmed by Sanger sequencing. gRNA sequences were as follows: *Rnf31*: TTCTCGTACGCACTGGCCCG, *Sharpin*: GTGGATCTTCAGTGAGACAT, and *Rbck1*: GTGGATCTTCAGTGAGACAT. *Ifnar1*^-/-^ iBMDMs were generously provided by Dr. Howard Young (NCI). *Trif*^*-/-*^, *Mavs*^-/-^, *Sting*^-/-^, and *Irf3*^-/-^ iBMDMs were generously provided by Dr. Kate Fitzgerald (UMass).

### Bacterial culture & cellular infection

Wt or dsRed+ *B*. *cenocepacia* strain J2315 (generously provided by Dr. David Greenberg [UTSW]) were streaked onto trypticase soy agar plates supplemented with 5% defibrinated sheep’s blood (Remel) and cultured for 72 h at 37°C. Colonies were then picked and inoculated into 25 mL of LB broth (Teknova) supplemented with 200 μg/mL chloramphenicol (for dsRed+ bacteria) and grown shaking at 37°C for 16–24 h. Bacteria were then diluted to the appropriate concentration, as determined by their OD_600nm_, in sterile PBS and washed twice. Bacteria were then added to wells containing cells and plates were centrifuged at 500 g for 5 min to synchronize infection and placed back in a 37°C incubator. This constitutes time zero for each infection. After 1 h, bacteria-containing media was replaced with complete DMEM supplemented with 250 μg/mL gentamicin and 500 μg/mL ceftazidime to kill any extracellular bacteria. After a further 2 h of incubation, media was replaced again with complete DMEM and cells were placed back in incubator for the duration of the infection. See below for how samples were processed for each endpoint assay.

GFP+ *Salmonella enterica subsp*. *enterica* serovar Typhimurium (*STm)* and wt *Burkholderia thailandensis* (*Bt*) were generously provided by Dr. Ed Miao (UNC). *STm* and *Bt* were stored on beads at -80°C. To grow these bacteria, a bead was added to 25 mL of LB broth and incubated at 37°C, shaking, for 16 h when they had reached stationary phase. Bacteria were then diluted to the appropriate concentration, as determined by their OD_600nm_, in sterile PBS and washed twice. Bacteria were then added to wells containing cells and plates were centrifuged at 500 g for 5 min to synchronize infection and placed back in a 37°C incubator. Cells were infected as they were with *Bc*, but with the following changes: Infection with *STm* was for 30 min before addition of 50 μg/mL of gentamicin for 30 min, followed by replacing the media with DMEM + 5 μg/mL gentamicin for the duration of the experiment. A similar procedure was followed for *Bt* infection, though bacteria was left to infect for 1 h before addition of 50 μg/mL gentamicin.

For experiments wherein BMDMs were infected with STm SL1344, BMDMs from both *Ifnar1*^-/-^ and WT C57/BL6 mice were differentiated for 5 days at 37°C in 5% CO_2_. Complete media containing DMEM (Corning CellGro) supplemented with 10% FBS (Gibco) and 100 mg/mL L-glutamine (Gibco) in the presence of 100 ng/mL of M-CSF (PeproTech) was used. Cells were harvested and replated at 7x10^4^ cells per well in a 24-well plate. Media was changed on day 6 and the cells were infected on day 7. *STm* SL1344 [75] and SL1344 ΔSPI2 [76] mCherry [77] were grown in LB-Miller plus streptomycin (100 μg/mL) or streptomycin and kanamycin (50 μg/mL), respectively, at 37°C with shaking at 225 rpm for 17 h. Subcultures in fresh media without antibiotic at 1:33 were grown for 3.5 h with shaking at 37°C. Bacteria were pelleted, washed in Hanks Balanced Salt Solution with Ca^2+^/Mg^2+^, and diluted 1:20 in media. Each well was infected at a MOI = 15 for 10 min at 37°C and 5% CO_2_. Wells were washed with HBSS 10 min p.i. and fresh media was added. At 30 min pi, media containing gentamicin (50 μg/mL) with or without/histidine (500 μg/mL, Sigma) was exchanged. At 45 min p.i. media was replaced with complete media plus 10 μg/mL gentamicin +/- histidine. Cells were imaged in an IncuCyte S3 imaging system (Essen Biosciences) with a 20X objective every 20 min for 24 h. Both phase and mCherry signal were monitored for 9 fields per well, duplicate wells per sample. The total mCherry signal was measured versus time.

### Endotoxic shock

Age-, sex-, and litter-matched mice were injected intra-peritoneally with 10 mg/kg body weight of lipopolysaccharide from *Salmonella enterica* serotype Minnesota (Sigma) diluted in sterile PBS. After injections, mice were weighed twice a day and euthanized at pre-determined endpoints. Survival curve data was analyzed by Log-Rank (Mantel-Cox) test.

### *In vivo* bacterial infection

Age-, sex-, and litter-matched mice were infected intranasally while under light gas anesthesia with 50 μL of dsRed+ J2315 at a dose of 5x10^6^ CFU/mouse in sterile PBS. For weight loss studies, the weight of the mice was measured every 12 h for up to 60 h, after which the mice were euthanized by CO_2_ inhalation. For bacterial load studies and cytokine measurement, mice were euthanized 18 h post-inoculation and brocho-alveolar lavages were performed using 1 mL of ice-cold PBS supplemented with 5% FBS and 2 mM EDTA. Samples were then spun down and supernatants were analyzed by a multi-analyte flow assay (LEGENDplex, Biolegend) while cells were resuspended in complete cell medium and allowed to adhere to a 96-well imaging plate for 2 h. After this time, the cells were fixed with 2% PFA for 10 min, then nuclei were stained with Hoescht 33342 nuclear dye (Molecular Probes). Intracellular bacteria were enumerated using the CellInsight NXT high-content imaging system (Thermo Scientific) as in the endpoint imaging described above.

### Quantitative PCR

Macrophages were infected with wt J2315 at an MOI of 1, or else stimulated with 5nm KLA, in a 48-well plate, with 50,000 cells/well in 200 μL antibiotic-free complete DMEM. After the appropriate infection time, cells were lysed in their plates with TriReagent (Zymo Research), then frozen at -80°C for at least 1 h and up to 3 days. RNA was extracted using the Zymo Direct-zol-96 RNA column-in-plate-based kit according to manufacturer’s directions. cDNA was produced using the iScript cDNA Synthesis Kit (Bio-Rad) according to manufacturer’s directions. cDNA was then analyzed using TaqMan primers/probes and the TaqMan Fast Advanced Master Mix (ThermoFisher) according to manufacturer’s directions on a QuantStudio 6 Real-Time PCR System (ThermoFisher). Relative quantification was performed using the ΔΔCt method, normalizing to *Hprt* expression and unstimulated control cells.

### Western blot

Differentiated BMDMs were lifted with 2mM EDTA, plated in 6-well format at 5x10^6^/well, and were allowed to adhere overnight. Cells were then infected J2315 as described above, at an MOI of 10. After antibiotic treatment, media was replaced with 1mL Optimem medium for the remainder of the experiment. Samples were collected at 4, 8, and 24 hr post infection by directly adding 4mL of cold Acetone to indicated wells followed by protein precipitation at -20°C overnight. Precipitates were then pelleted at 2000 g in polypropylene tubes and the acetone was aspirated. Pellets were immediately resuspended in 60uL 2x SDS Sample buffer followed by heating at 95°C for 10 min. Samples were run on 12% Bis-tris gels in MES running buffer followed by transfer onto methanol-activated PVDF membrane via wet-transfer. Blots were blocked in 2% BSA + 0.05% Tween-20 TBS and were probed for Caspase1 (Abnova Casper1) and Gsdmd (Abcam) 1:500 overnight at 4°C. Blots were probed with appropriate HRP-conjugated secondaries and were developed with Dura ECL (Thermo) in a BioRad Chemidoc.

### ELISA

IL1β was quantitated using R&D System murine Duoset Elisa reagents according to manufacturer protocols. Briefly, infections were performed as biological quadruplicates and supernatants were collected at indicated timepoints. 10 uM Nigericin (Cayman) and 1 mM LeuLeuOMe (ChemImpex) were used as canonical inflammasome triggers. Capture antibody was applied to 384-well Immunisorp plates followed by blocking in 2% BSA-PBS. Supernatants were applied to blocked wells for 2 h. Detection antibody and HRP-Streptavidin was applied sequentially for 1 hour each. Plates were washed thoroughly four times with 0.05% Tween-20 PBS. ELISAs were developed with 1-step TMB for roughly 30 min followed by a stop solution spike. Absorbances were collected with a BMG Omegastar and values within the linear range of the assay were interpolated for graphing.

### Plating-based bacterial replication assays

Cells were infected with wt J2315 at an MOI of 1 in a 96-well plate, with 25,000 cells/well in 100μL antibiotic-free complete DMEM. At the appropriate time, media was removed from infected cells and wells were washed 3x with warm, sterile PBS. Cells were then lysed in 100 μL of H_2_O for 10 min at RT. Lysates were then serially diluted 1:10 in sterile PBS down to a 10^−6^ dilution. These dilutions were then plated on tryptic soy agar plates supplemented with 5% sheep’s blood and allowed to grow overnight at 37°C. Colonies were counted and counts were converted to their original concentrations from the cell lysate. For IFN pre-stimulation assays, 1000 U/mL recombinant IFN-β (PBL Assay Science) or 100 ng/mL recombinant IFN-γ (PBL Assay Science) was added for the given times before the removal of IFN-containing media and replacement with media containing J2315. Infections then proceeded as explained above.

### High-content imaging

Cells were infected with dsRed-expressing J2315 or GFP+ *STm* at an MOI of 1 in a Falcon black-walled 96-well imaging plate (Fisher Scientific), with 25,000 cells/well in 100 μL of antibiotic-free complete DMEM. After 1 h, media was replaced with complete DMEM containing gentamicin and ceftazidime, as above, for 2 h. For live-cell imaging, cells were stained with 1ug/mL Hoechst 33342 (ThermoFisher) in phenol-red-free complete DMEM and, after the removal of antibiotics at 3 h pi, 1uM Draq7 in phenol-red-free complete DMEM was added if indicated. Plates were loaded onto the CellInsight CX7 imaging system (ThermoFisher) and imaged every 30 min for 18–24 h under low-intensity illumination (1–5% per channel) using a 20x objective. Cells were kept in an on-stage incubator providing 37°C temperature, 5% CO_2_, and 55% relative humidity throughout imaging. For endpoint imaging, antibiotics were removed at 3 h pi, and the infection was allowed to continue in the incubator for 22 h, after which cells were fixed with 2% PFA for 10 min at RT. Cells were then permeabilized with 0.1% Triton X-100 in sterile PBS for 10 min at RT, then blocked in 5% BSA for 1 h. For LC3b staining, cells were fixed in cold methanol for 10 min followed by rehydration in PBS prior to blocking. Antibody staining for J2315, LC3B (CST/Nanotools 2G6) or total ubiquitin (CST) was performed overnight at 4°C. Secondary staining and nuclear staining with Hoechst 33342 were performed before loading the plate onto the CellInsight NXT imaging system (ThermoFisher). In all cases, cells were counted using the nuclear counterstain and the bacteria-containing area was enumerated based on dsRed expression. Bacterial spot area was only counted if it was found within 10 pixels of a live cell nucleus.

### Flow cytometry

Cells were infected as in the RT-PCR experiments with dsRed+ J2315, but infection was allowed to continue for 22 h. Cells were lifted from their wells using 100 μL of TrypLE Express enzyme (Gibco) for 10min at 37°C followed by vigorous pipetting. Cells were spun down, then fixed in 2% PFA for 10min at RT before three washes in sterile PBS. Cells were run on a FACSCanto flow cytometer (BD) and analyzed using FlowJo. Intact cells were gated using FSC-A and SSC-A parameters after which dsRed fluorescence was used as a proxy for bacterial load.

### Cytokine quantification

Supernatants were collected from infected cells at given time points and cytokine concentrations were analyzed using the LEGENDplex mouse anti-virus response panel (Biolegend) according to manufacturer’s directions. Samples were run on a FACSCanto flow cytometer and analyzed with FlowJo.

### siRNA knockdown

THP1 cells were transfected with siRNA using a previously established protocol [78]. The following siRNA were used; human LGALS3BP: Ambion, Cat# s8152; human SQSTM1 (p62): Ambion, Cat# s16961; non-targeting control (NTC) siRNA: Dharmacon, Cat# D-001210-05-05. Knockdown efficiency was assessed by qPCR using TaqMan Gene Expression probe sets (ThermoFisher) following the manufacturers recommended protocol: human LGALS3BP, Cat# 4331182; human SQSTM1 (p62), Cat# 4331182.

### RNAseq (and analysis)

BMDMs from WT and *Ifnar1*^-/-^ mice were infected with J2315 as above or stimulated with 5nM of KLA for the given amounts of time before lysis in TriReagent (Zymo). RNA was quantified and integrity was analyzed using a Bioanalyzer High Sensitivity RNA Analysis kit (Agilent). Sequencing libraries were produced using the TruSeq Stranded mRNA kit and TruSeq Single Indexes (Illumina) according to manufacturer’s directions and sequenced on a NextSeq 500 to generate greater than 50 million reads per library. Raw fastq files were then trimmed for quality and adapter contamination using *Cutadapt v2*.*10* [79] and trimmed reads were mapped to the mm10 mouse reference genome and Gencode M25 transcriptome using *STAR v2*.*5*.*3* [80] in 2-pass mode. Gene-level expression quantification was performed using *RSEM v1*.*3*.*0* [81] and standard differential expression was performed using the R package *limma* with voom normalization [82]. Prior to differential expression and downstream timecourse analysis, genes were filtered that had <1 counts per million (CPM) across <2 samples. Pairwise differential expression analysis was performed using log_2_ fold-change values from *limma*. For the heat map in Fig 4A, genes where log_2_ FC was either > 1 or < -1.5 and FDR < 0.05 were included. The heat map was created using Multiple Experiment Viewer. For the timecourse expression analysis, we used the R package *maSigPro* [39] and all pathway enrichment analyses used the Reactome database and over-representation analysis (ORA) implemented in *WebGestalt* [83] at an FDR < 0.05.

### Glucose 6-phosphate dehydrogenase assay

WT and *Ifnar1*^-/-^ iBMDMs were infected with either dsRed+ J2315 or GFP+ *STm* at an MOI of 1 for 22h, as in all bacterial replication assays. Cell death was measured by glucose 6-phosphate dehydrogenase release using the Vybrant Cytotoxicity Assay Kit (Molecular Probes) according to manufacturer’s directions.

### Seahorse assay

The glycolytic rate assay (GRA) was performed using the XF96 Seahorse Metabolic Analyzer (Agilent Biosciences) according to manufacturer’s protocol. Briefly, 1x10^5^ WT or *Ifnar1*^-/-^ iBMDMs were cultured in 200 μl of complete media a day prior to GRA. On the day of the assay, media was replaced with GRA medium (Seahorse XF DMEM, pH7.4 supplemented with 10 mM glucose, 2 mM L-Glutamine and 1 mM Sodium-Pyruvate (Agilent Biosciences)), and the cells were incubated at 37°C in a non-CO_2_ incubator for one hour. Basal levels were measured thrice followed by live or heat-killed *Bc* injection at MOI 10. Proton efflux rate (PER) levels were calculated every 5 minutes for 7.5 hours. Data are represented as the fold change relative to uninfected cells.

### H_2_O_2_ quantification

WT and *Ifnar1*^-/-^ iBMDMs were either infected with wt J2315 at an MOI of 10, as above, stimulated with heat-killed wt J2315 at the equivalent of an MOI of 10, or stimulated with 1000 U/mL of recombinant mouse IFNβ (PBL Assay Science) for 24 h. H_2_O_2_ quantification was performed using the ROS Glo H2O2 Assay kit (Promega) according to manufacturer’s directions, adding the H_2_O_2_ substrate solution for the final 6 h of infection/stimulation. Luminescence was quantified using a FLUOstar microplate reader (BMG Labtech).

### Statistics

All statistical analyses outside of the RNAseq analysis were performed using Prism 8 (GraphPad). Unless otherwise noted, Mann-Whitney U tests were used to compare groups. For all analyses: * = p≤0.05, ** = p≤0.01, *** = p≤0.001, and **** = p≤0.0001. All error bars shown are mean +/- SEM.

## Supporting information

S1 Fig A, B) WT (JAX 664) and *Ifnar1*-/- mice were injected with 10mg/kg LPS, as in Fig 1C, and survival was tracked over 5 days. A) presents three separate experiments (n ≥ 5 per genotype) while B) shows these three experiments combined. C) Litter-matched WT and *Ifnar1*-/- mice were infected with 5x10^6^ CFUs of wt J2315, as in Fig 1D. Broncho-alveolar lavages were performed and cytokines were measured using a LegendPLEX anti-viral cytokine assay.
(TIF)

S2 Fig A) WT and *Ifnar1*^-/-^ iBMDMs were infected with GFP+ *Burkholderia thailandensis*, as in Fig 2D, for 22 h. Bacterial replication was measured by mean GFP fluorescence in the infected population. B, C, D) WT and *Ifnar1*^-/-^ iBMDMs were infected with dsRed+ *Bc*, as in Fig 2F and 2B) The total number of cells imaged, C) the total spot area per cell, and D) the number of live cells per imaged field are enumerated. All experiments were performed with n ≥ 3, and are shown as representative of 2 independent experiments. * = p≤0.05 and *** = p≤0.001 by Mann-Whitney U test.
(TIF)

S3 Fig A, B) WT and *Ifnar1*^-/-^ iBMDMs were infected with A) dsRed+ *Bc* (MOI = 1) or B) GFP+ *STm* (MOI = 10) for 22 h. Supernatants were removed, and levels of glucose 6-phosphate dehydrogenase were measured. Percent cell lysis was enumerated as compared to uninfected cells lysed with lysis buffer. C, D) WT, *Casp1/11*-/-, and *Ifnar1*-/- BMDMs were infected with wt J2315 for the indicated times before supernatants were removed and cells were lysed. C) Lysates were analyzed by Western blot for Caspase-1, Gasdermin-D, GAPDH, and RhoGDI (loading control). D) IL-1β was measured in supernatants by ELISA. 1 mM Leu-Leu-O-methyl (LLoM) and 10 μM nigericin (Nig) were used as positive controls of inflammasome activation. E) WT, *Casp1/11*-/-, and *Ifnar1*-/- BMDMs were infected with wt J2315 and then live-imaged in the presence of Draq7 after 3 h of infection. Draq7 uptake was measured for 19 h. *** = p≤0.001, **** = p≤0.0001 by Mann-Whitney U test.
(TIF)

S4 Fig A) A time point-resolved view of Fig 4B, showing the number of genes that have reached log_2_ fold-change > 1, FDR ≤ 0.05 at each specific time point. B) The number of genes for which the fold-change in WT cells was at least 2x greater than the fold-change in *Ifnar1*^-/-^ cells at any point in the time course. C) Top 10 pathways associated with the Cluster 3 gene members in *Bc*-infected cells, as found by Reactome pathway analysis.
(TIF)

S5 Fig A) WT and *Ifnar1*^-/-^ iBMDMs were infected with live or heat-killed *Bc* at an MOI of 10 while oxygen consumption and the pH of the culture media was measured on a Seahorse XF analyzer. Proton efflux rate was enumerated over the course of 7.5 h. B, C) WT and *Ifnar1*^-/-^ iBMDMs were infected with live or heat-killed *Bc* at given MOIs or else stimulated with 1000 U/mL of rIFNβ for 24 h. H_2_O_2_ was measured using a luminescence-based assay. B) shows luminescence levels while C) shows luminescence relative to unstimulated cells. All experiments were performed with n = 5, and are shown as representative of 2 independent experiments *** = p≤0.001 by Mann-Whitney U test.
(TIF)

S6 Fig A) WT, *Rnf31*+/-, *Rbck1*-/-, and *Sharpin*-/- RAW264.7 cells were infected with wt J2315, as in Fig 5. After 20h, cells were lysed and lysates were cultured for 24 h before enumerating colonies; n = 5 per condition, representative of 3 independent experiments. B) The same cell lines were pre-stimulated with rIFNβ or PBS for 24 h before infecting with dsRed+ J2315 (MOI = 1). Bacterial growth was measured using live-cell high-content imaging based on dsRed fluorescence. Data presented as fold-change in dsRed fluorescence intensity compared to t = 3 h p.i. C) RT-PCR showing effective knockdown of LGALS3BP or p62 in Fig 5F. mRNA is shown relative to cells transfected with a non-target control (NTC) siRNA. * = p≤0.05, ** = p≤0.01, *** = p≤0.001, and **** = p≤0.0001 by Mann-Whitney U test.
(TIF)

S7 Fig WT, *Trif*-/-, *Sting*-/-, and *Mavs*-/- iBMDMs were infected with J2315 (MOI = 1), fixed at given time points, and stained for LC3b. A) Autophagosome formation was measured by LC3b puncta formation and is presented relative to uninfected cells of each line. B) Representative images of WT, *Trif*-/-, and *Sting*-/- iBMDMs at 16 h p.i. showing LC3b (green in merged image) and J2315 (red in merged image) spot formation.
(TIF)

S1 Movie WT (left) and *Rnf31*^+/-^ (right) RAW264.7 cells were infected with dsRed+ *Bc*, as in Fig 5D. This movie shows dsRed fluorescence and nuclear staining every 30 min from t = 3 h until t = 22.5 h.
(MP4)

S2 Movie WT (left) and *Ifnar1*^-/-^ (right) iBMDMs were infected with dsRed+ *Bc*, as in Fig 6C. This movie shows dsRed fluorescence and nuclear staining every 30 min from t = 3 h until t = 23.5 h.
(MP4)

S1 Raw Data This spreadsheet contains the raw data used to generate the figures in the manuscript. Raw RNAseq data was deposited in GEO, reference #GSE165020.
(XLSX)
