# Correction: Type I IFNs facilitate innate immune control of the opportunistic bacteria Burkholderia cenocepacia in the macrophage cytosol

**DOI:** 10.1371/journal.ppat.1009821

**Published:** 2021-08-04

**Authors:** Michael G. Dorrington, Clinton J. Bradfield, Justin B. Lack, Bin Lin, Sinu P. John, Jonathan J. Liang, Tregei Starr, Orna Ernst, Julia L. Gross, Jing Sun, Alexandra H. Miller, Olivia Steele-Mortimer, Iain D. C. Fraser

Sinu P. John should be included in the author byline. He should be listed as fifth author, and his affiliation is 1: Signaling Systems Section, Laboratory of Immune System Biology, NIAID, NIH, Bethesda, Maryland, United States of America. The contributions of this author are as follows: Investigation, Formal Analysis.

The correct citation is: Dorrington MG, Bradfield CJ, Lack JB, Lin B, John SP, Liang JJ, et al. (2021) Type I IFNs facilitate innate immune control of the opportunistic bacteria Burkholderia cenocepacia in the macrophage cytosol. PLoS Pathog 17(3): e1009395. https://doi.org/10.1371/journal.ppat.1009395
