# Supplementary figures and images for: Type I IFNs facilitate innate immune control of the opportunistic bacteria Burkholderia cenocepacia in the macrophage cytosol
Source: PLoS Pathog. 2021 Mar 8;17(3):e1009395. doi: 10.1371/journal.ppat.1009395 (PMC7971856; doi:10.1371/journal.ppat.1009395)

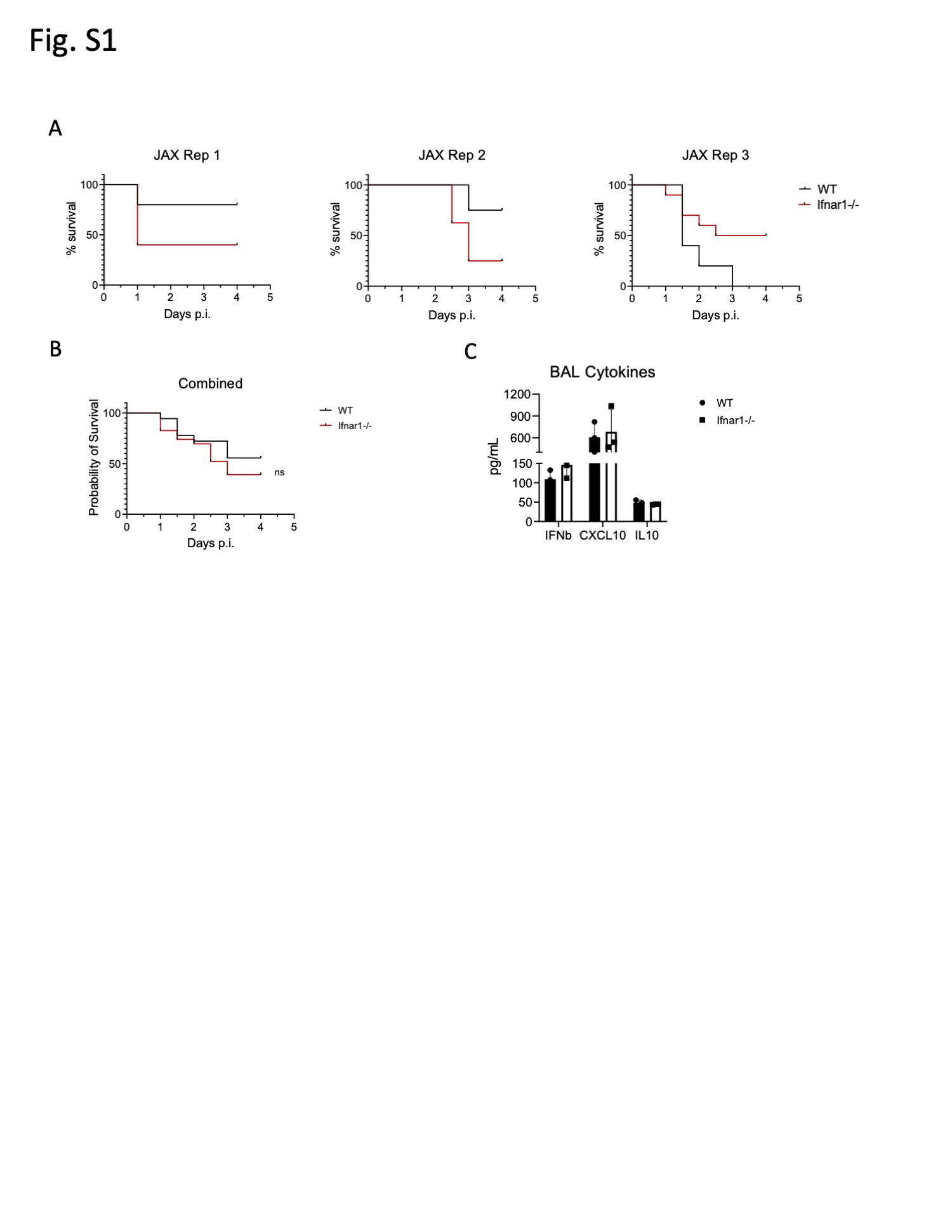

Supplement: S1 Fig — A, B) WT (JAX 664) and Ifnar1-/- mice were injected with 10mg/kg LPS, as in Fig 1C, and survival was tracked over 5 days. A) presents three separate experiments (n ≥ 5 per genotype) while B) shows these three experiments combined. C) Litter-matched WT and Ifnar1-/- mice were infected with 5x10^6^ CFUs of wt J2315, as in Fig 1D. Broncho-alveolar lavages were performed and cytokines were measured using a LegendPLEX anti-viral cytokine assay. (TIF) [file ppat.1009395.s001.tif]

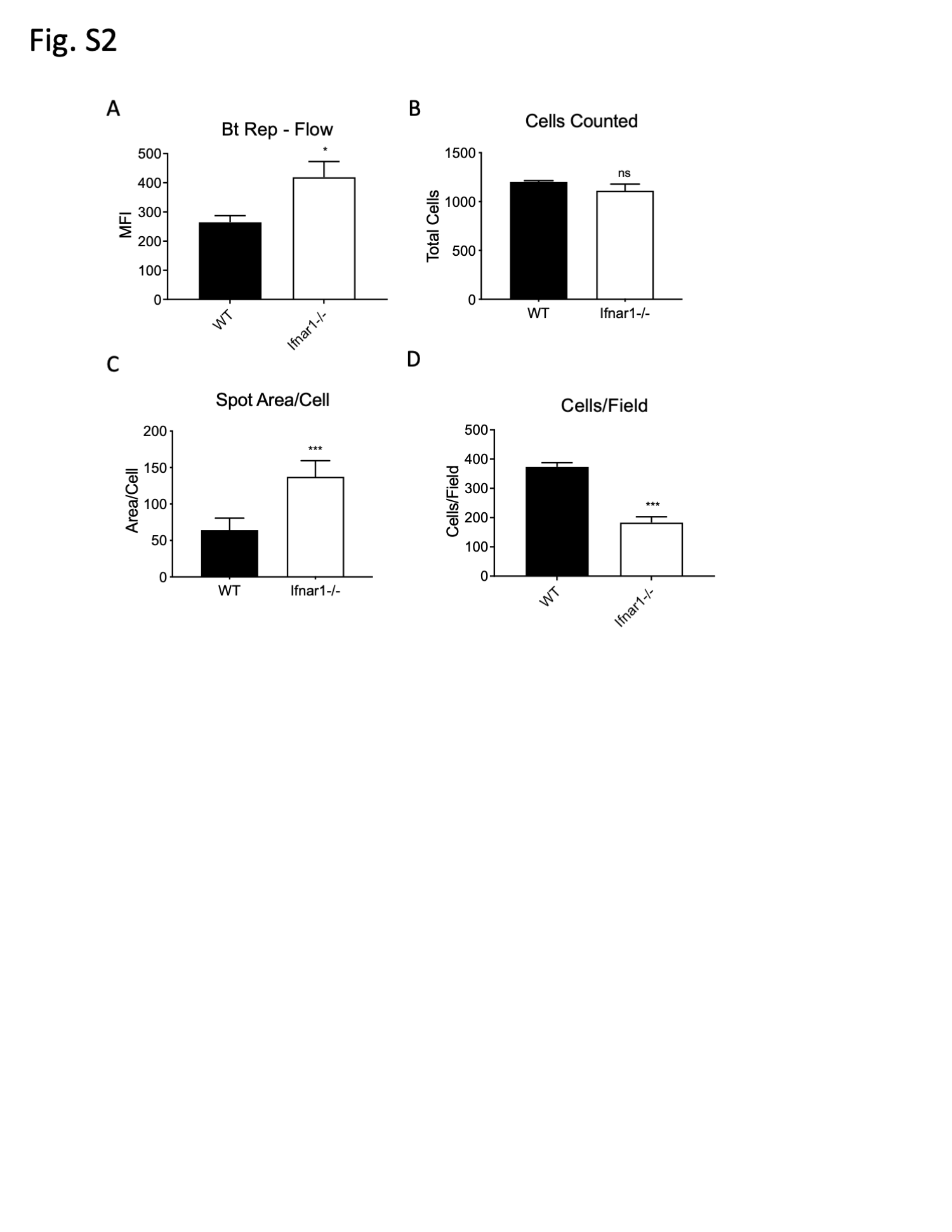

Supplement: S2 Fig — A) WT and Ifnar1-/- iBMDMs were infected with GFP+ Burkholderia thailandensis, as in Fig 2D, for 22 h. Bacterial replication was measured by mean GFP fluorescence in the infected population. B, C, D) WT and Ifnar1-/- iBMDMs were infected with dsRed+ Bc, as in Fig 2F and 2B) The total number of cells imaged, C) the total spot area per cell, and D) the number of live cells per imaged field are enumerated. All experiments were performed with n ≥ 3, and are shown as representative of 2 independent experiments. * = p≤0.05 and *** = p≤0.001 by Mann-Whitney U test. (TIF) [file ppat.1009395.s002.tif]

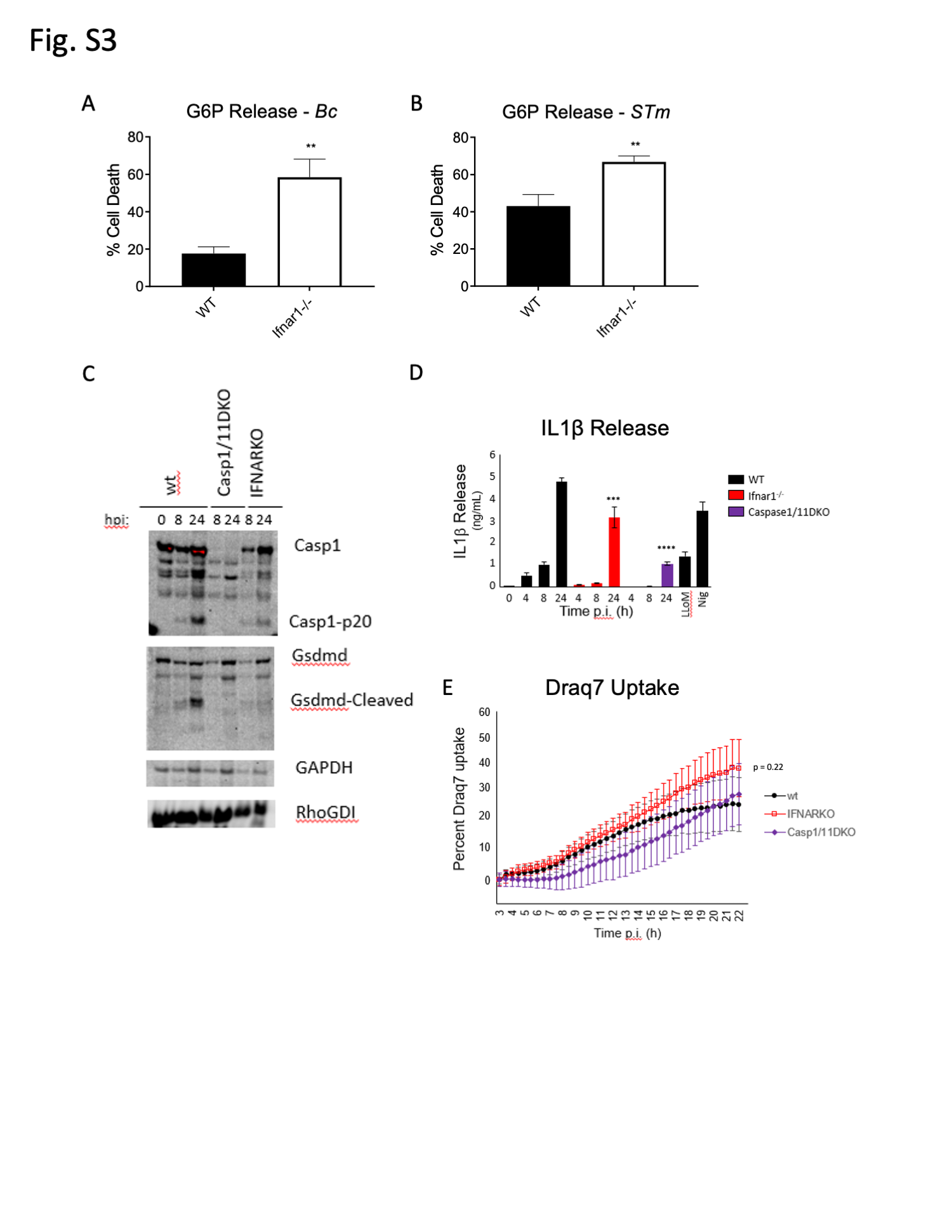

Supplement: S3 Fig — A, B) WT and Ifnar1-/- iBMDMs were infected with A) dsRed+ Bc (MOI = 1) or B) GFP+ STm (MOI = 10) for 22 h. Supernatants were removed, and levels of glucose 6-phosphate dehydrogenase were measured. Percent cell lysis was enumerated as compared to uninfected cells lysed with lysis buffer. C, D) WT, Casp1/11-/-, and Ifnar1-/- BMDMs were infected with wt J2315 for the indicated times before supernatants were removed and cells were lysed. C) Lysates were analyzed by Western blot for Caspase-1, Gasdermin-D, GAPDH, and RhoGDI (loading control). D) IL-1β was measured in supernatants by ELISA. 1 mM Leu-Leu-O-methyl (LLoM) and 10 μM nigericin (Nig) were used as positive controls of inflammasome activation. E) WT, Casp1/11-/-, and Ifnar1-/- BMDMs were infected with wt J2315 and then live-imaged in the presence of Draq7 after 3 h of infection. Draq7 uptake was measured for 19 h. *** = p≤0.001, **** = p≤0.0001 by Mann-Whitney U test. (TIF) [file ppat.1009395.s003.tif]

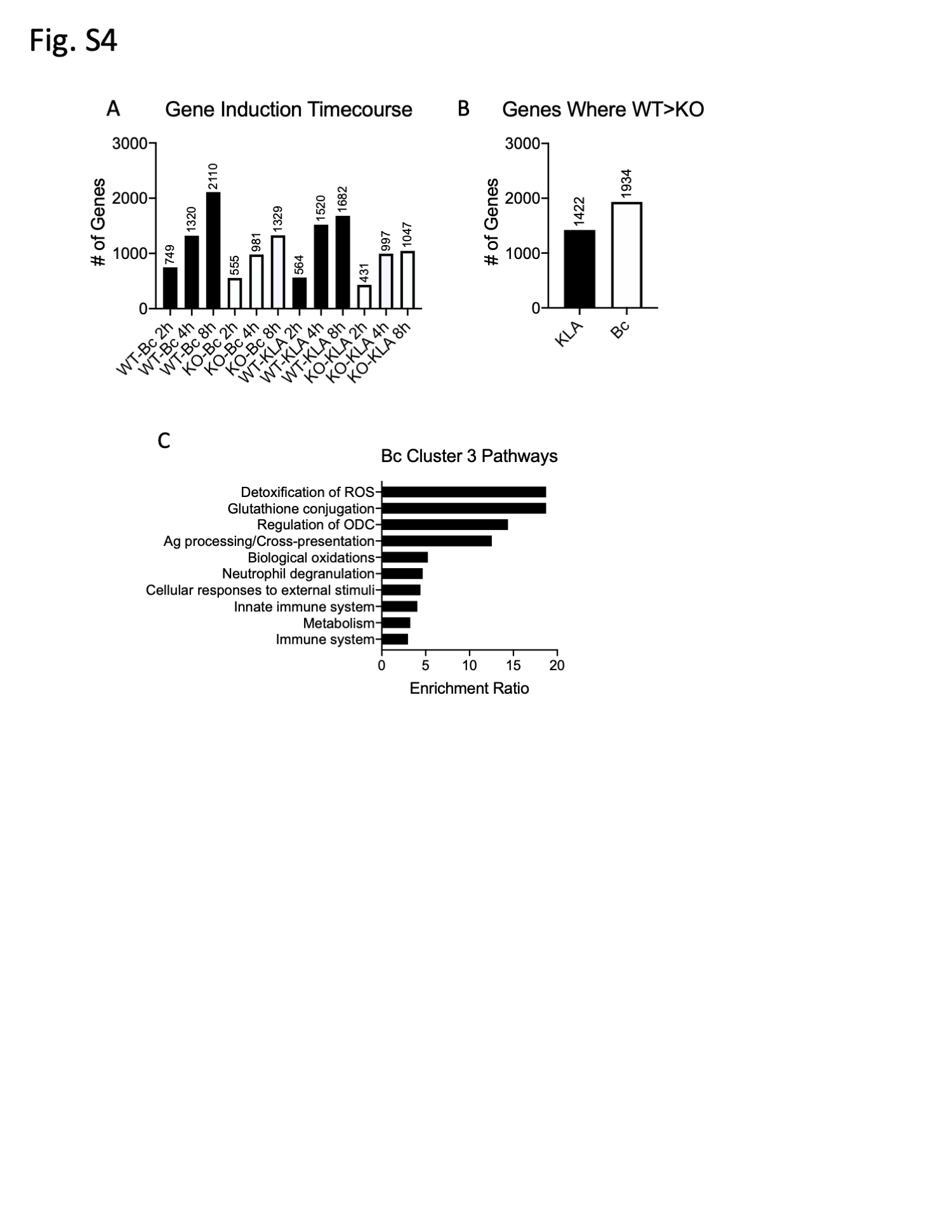

Supplement: S4 Fig — A) A time point-resolved view of Fig 4B, showing the number of genes that have reached log2 fold-change > 1, FDR ≤ 0.05 at each specific time point. B) The number of genes for which the fold-change in WT cells was at least 2x greater than the fold-change in Ifnar1-/- cells at any point in the time course. C) Top 10 pathways associated with the Cluster 3 gene members in Bc-infected cells, as found by Reactome pathway analysis. (TIF) [file ppat.1009395.s004.tif]

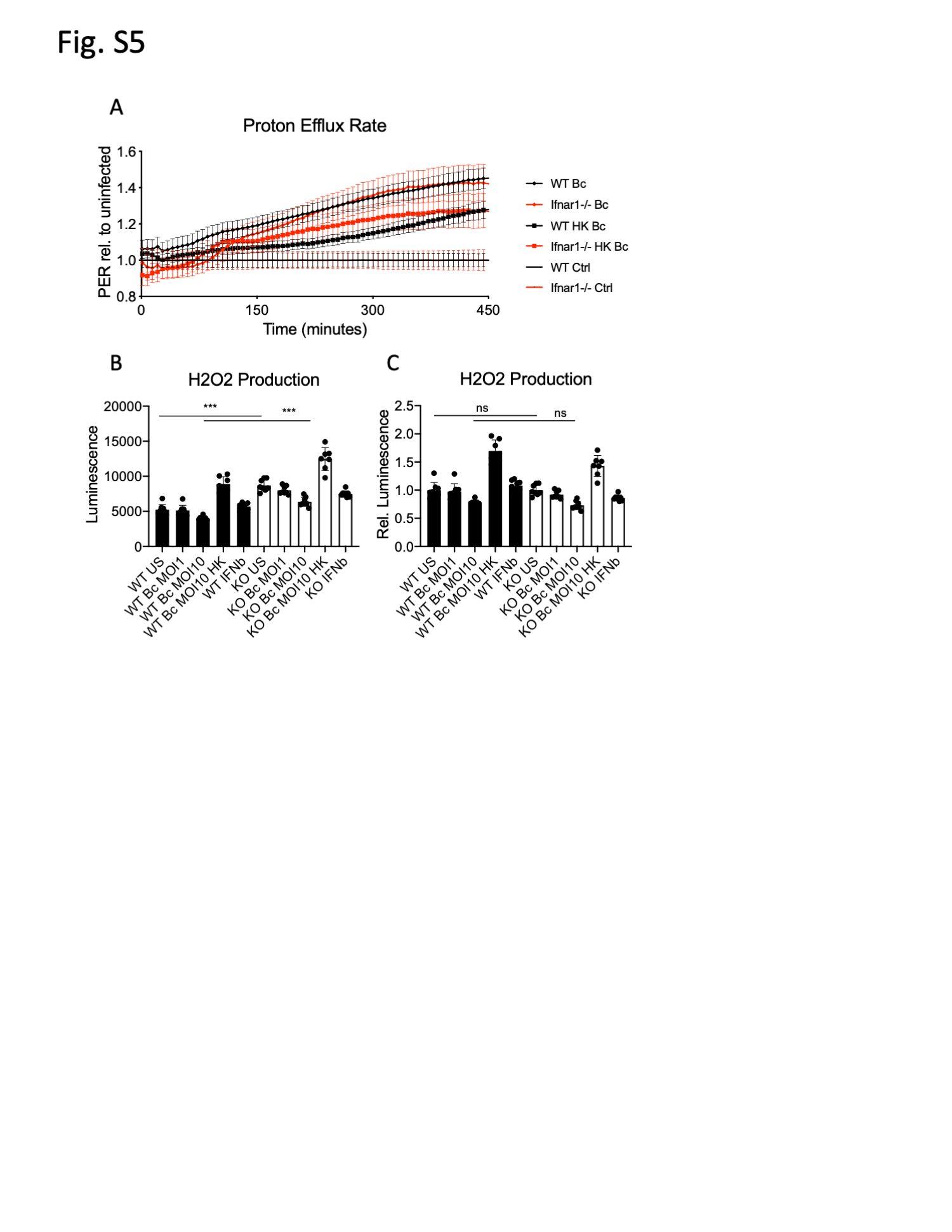

Supplement: S5 Fig — A) WT and Ifnar1-/- iBMDMs were infected with live or heat-killed Bc at an MOI of 10 while oxygen consumption and the pH of the culture media was measured on a Seahorse XF analyzer. Proton efflux rate was enumerated over the course of 7.5 h. B, C) WT and Ifnar1-/- iBMDMs were infected with live or heat-killed Bc at given MOIs or else stimulated with 1000 U/mL of rIFNβ for 24 h. H2O2 was measured using a luminescence-based assay. B) shows luminescence levels while C) shows luminescence relative to unstimulated cells. All experiments were performed with n = 5, and are shown as representative of 2 independent experiments *** = p≤0.001 by Mann-Whitney U test. (TIF) [file ppat.1009395.s005.tif]

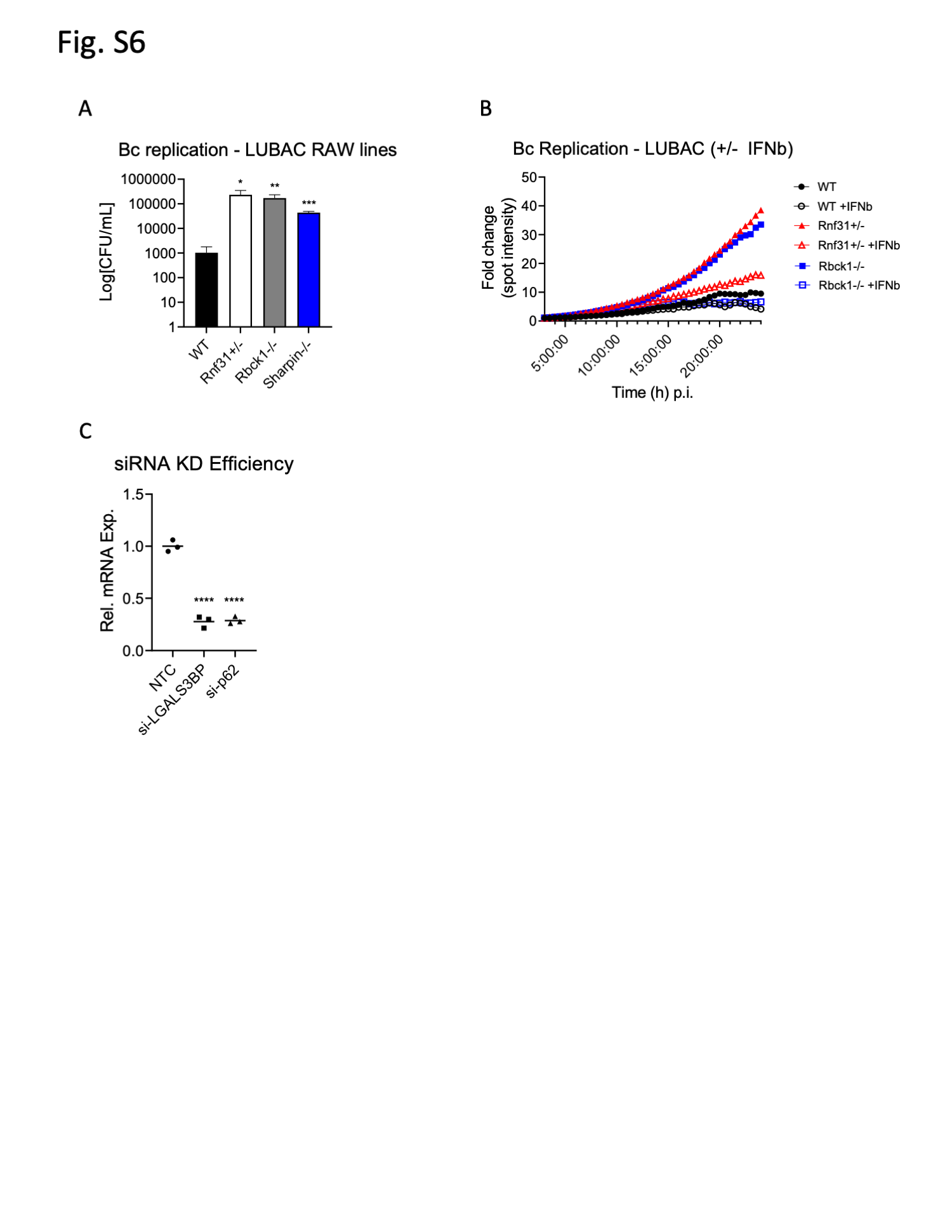

Supplement: S6 Fig — A) WT, Rnf31+/-, Rbck1-/-, and Sharpin-/- RAW264.7 cells were infected with wt J2315, as in Fig 5. After 20h, cells were lysed and lysates were cultured for 24 h before enumerating colonies; n = 5 per condition, representative of 3 independent experiments. B) The same cell lines were pre-stimulated with rIFNβ or PBS for 24 h before infecting with dsRed+ J2315 (MOI = 1). Bacterial growth was measured using live-cell high-content imaging based on dsRed fluorescence. Data presented as fold-change in dsRed fluorescence intensity compared to t = 3 h p.i. C) RT-PCR showing effective knockdown of LGALS3BP or p62 in Fig 5F. mRNA is shown relative to cells transfected with a non-target control (NTC) siRNA. * = p≤0.05, ** = p≤0.01, *** = p≤0.001, and **** = p≤0.0001 by Mann-Whitney U test. (TIF) [file ppat.1009395.s006.tif]

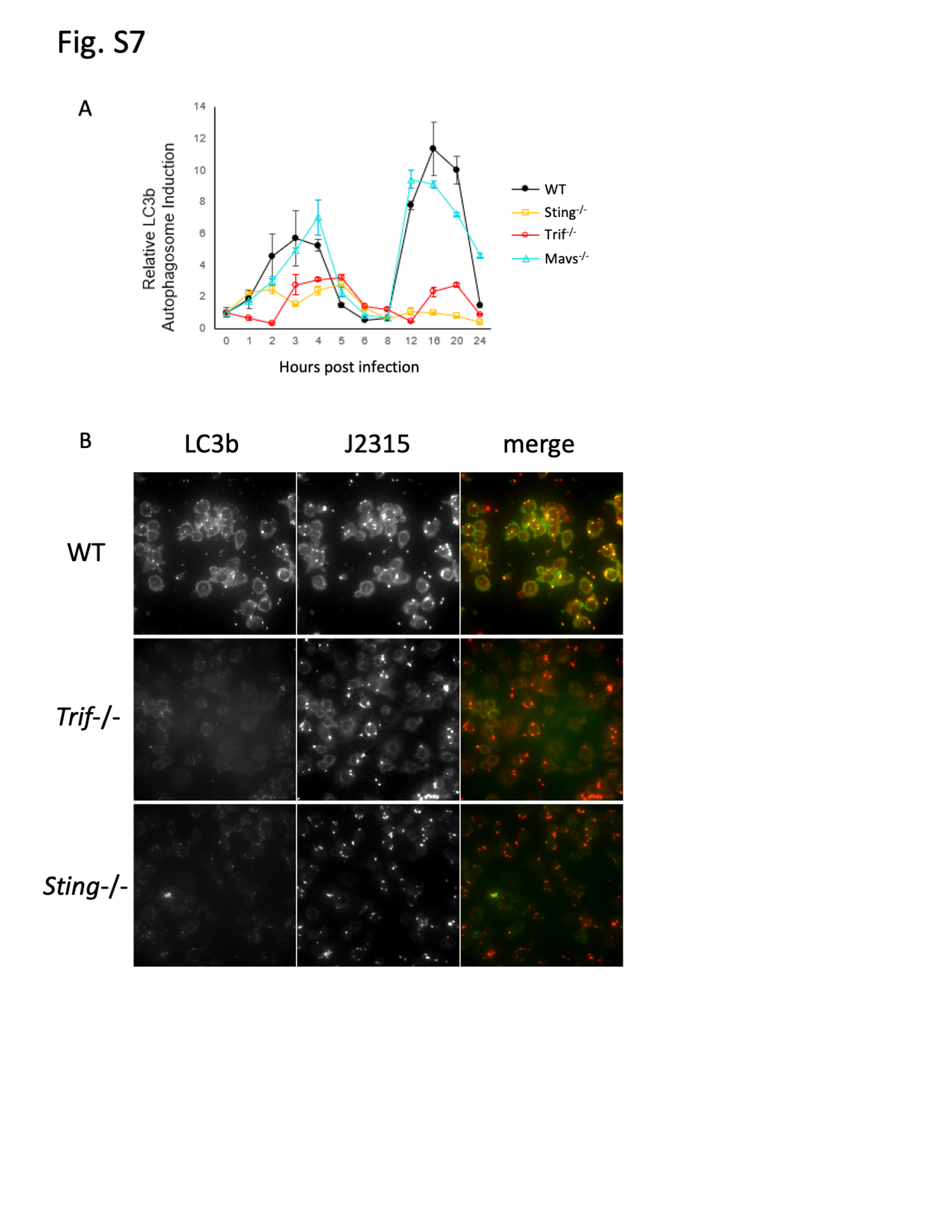

Supplement: S7 Fig — WT, Trif-/-, Sting-/-, and Mavs-/- iBMDMs were infected with J2315 (MOI = 1), fixed at given time points, and stained for LC3b. A) Autophagosome formation was measured by LC3b puncta formation and is presented relative to uninfected cells of each line. B) Representative images of WT, Trif-/-, and Sting-/- iBMDMs at 16 h p.i. showing LC3b (green in merged image) and J2315 (red in merged image) spot formation. (TIF) [file ppat.1009395.s007.tif]
